# Algorithm dependence of patient phenotypes in Long COVID: a patient-led, multi-method clustering of 6031 patients using 162 self-reported symptoms

**DOI:** 10.1093/oxfimm/iqag010

**Published:** 2026-06-13

**Authors:** Tessa D Green, Chris McWilliams, Leonardo de Figueiredo, Letícia Soares, Beth Pollack, Alison K Cohen, Tan Zhi-Xuan, Tess Falor, Hannah E Davis

**Affiliations:** Patient-Led Research Collaborative, Calabasas, CA, 91302, United States; Department of Systems Biology, Harvard Medical School, Boston, MA, 02115, United States; Patient-Led Research Collaborative, Calabasas, CA, 91302, United States; Department of Engineering Mathematics, University of Bristol, Bristol, BS8 1QU, United Kingdom; Patient-Led Research Collaborative, Calabasas, CA, 91302, United States; Patient-Led Research Collaborative, Calabasas, CA, 91302, United States; Patient-Led Research Collaborative, Calabasas, CA, 91302, United States; Department of Biological Engineering, Massachusetts Institute of Technology, Cambridge, MA, 02139, United States; Patient-Led Research Collaborative, Calabasas, CA, 91302, United States; Department of Epidemiology & Biostatistics and Philip R. Lee Institute for Health Policy Studies, University of CA San Francisco, San Francisco, CA, 94143, United States; Department of Electrical Engineering and Computer Science, Massachusetts Institute of Technology, Cambridge, MA, 02139, United States; Department of Computer Science, National University of Singapore, Singapore, 117417, Singapore; Patient-Led Research Collaborative, Calabasas, CA, 91302, United States; Patient-Led Research Collaborative, Calabasas, CA, 91302, United States

## Abstract

**Objectives:**

Long COVID, characterized by symptoms that remain or emerge in the months after acute COVID-19 infection, is a multisystemic condition with highly variable patient presentations. Phenotyping studies have reported divergent symptom clusters, increasingly used to design trials and interpret biomarker data. However, robustness of these clusters across analytic methods remains uncertain.

**Methods:**

We analyzed data from 6 031 adults with ≥ 90 days of illness from a patient-led international survey. Participants reported presence/absence of 162 symptoms, post-exertional malaise severity and demographics. We applied three unsupervised machine learning approaches to the same symptom matrix, evaluating the resulting clusterings for concordance, robustness to subsampling, and relationship to symptom burden, post-exertional malaise severity, age and gender.

**Results:**

Each method produced clinically plausible symptom clusters, but concordance across methods was low. All three approaches identified a high-symptom-burden group enriched for post-exertional malaise severity, and lower-symptom-burden groups with older mean age and a lower proportion of women. Symptom count consistently correlated with higher post-exertional malaise severity and a greater proportion of women. Manifold analysis revealed that the overall symptom space was largely continuous, lacking clear cluster boundaries.

**Conclusions:**

The strong dependence of patient clusters on algorithm choice suggests that single-method Long COVID phenotyping may produce incomplete or unstable subgroup definitions. Clustering methods may impose artificial boundaries on a smoothly varying symptom landscape, especially in studies capturing fewer symptoms. Phenotyping efforts should assess clustering robustness and avoid overinterpreting single-method results. Our multi-method analysis highlights the importance of considering the full breadth of patient symptoms when evaluating treatments.

## Introduction

Long COVID is a debilitating multisystemic condition with substantial global prevalence and long-term health impact. Recent reviews estimate that millions of people continue to experience persistent multi-systemic symptoms months to years after infection, with profound effects on function, employment, and quality of life [1–3]. Mechanistically, Long COVID appears to reflect multiple, overlapping biological processes, including vascular endothelial dysfunction [4, 5], fibrinaloid microclots [6], persisting reservoirs of SARS-CoV-2 [7–9], autoimmunity [10], T-cell dysregulation and exhaustion [11–13], reactivation of latent viral infections [11], organ damage [14], gut dysbiosis [15, 16], and neuroinflammation [17]. These pathophysiological mechanisms are accompanied by persistent inflammatory and immunologic abnormalities that can remain detectable for months to years after acute infection [18]. Together, this complexity produces a wide range of symptoms; here, we investigate how these symptoms may cluster into clinically meaningful phenotypes.

Several large patient-led and clinical cohort studies have documented the breadth of symptoms in Long COVID, including a 3762-person patient-led survey identifying over 200 symptoms across 10 organ systems [19] and a deep phenotyping using the Human Phenotype Ontology [20] identifying 287 phenotypic abnormalities associated with Long COVID. Among these symptoms, post-exertional malaise (PEM), a physiological state characterized by worsened symptoms after disproportionately minor physical or cognitive exertion, has been identified as common in certain Long COVID cohorts and phenotypes, and has been associated with greater functional impairment and disability when explicitly assessed [21, 22]. However, the reported prevalence and clinical prominence of PEM varies substantially across studies depending on sampling strategies, symptom definitions, and the scope of symptom assessment, and remains incompletely characterized in the broader population of individuals following infection [23]. In the patient-led survey used here, PEM was explicitly assessed as a symptom of interest, including separate self-reported severity ratings, but the survey did not capture key defining features such as delay, duration, or disproportionate response to minimal exertion, and thus may include a broader range of exertion-related symptom exacerbations. As such, while we treat PEM as described in this survey as a key interpretive dimension of the symptom phenotypes identified, it should not be assumed to be directly comparable to clinically defined PEM or to be uniformly present across all Long COVID populations.

In parallel, a growing body of work has used clustering and related unsupervised methods to define Long COVID “phenotypes” or symptom clusters in both clinical and population-based cohorts. Approaches include clustering using self-reported symptom lists in outpatient and post-hospital cohorts [24–27], high-dimensional analyses of electronic health records [28], and longitudinal studies of symptom trajectories [29]. Systematic reviews highlight substantial heterogeneity in both clustering methods and the reported phenotypes, and vary in sampling frame, data source (survey versus EHR), and involvement of patient experts [30, 31]. Many primary clustering studies also rely on relatively short symptom lists, though this is not systematically quantified in existing reviews.

These phenotypes are increasingly being used to stratify patients in clinical trials, interpret biomarker studies, and communicate with patients and clinicians. Yet Long COVID is characterized by overlapping symptomology and likely overlapping endotypes, raising questions about whether phenotypes derived from a single clustering method on a limited symptom set are robust enough to support clinical decision making or regulatory use. Moreover, clustering algorithms differ in their assumptions about data structure, noise, and cluster shape; if cluster assignments are highly sensitive to algorithm choice, then single-method solutions may be unstable or misleading.

In this context, patient-led research provides a complementary perspective. The Patient-Led Research Collaborative (PLRC) survey was designed and conducted by people with Long COVID, emphasizing comprehensive symptom coverage and lived experience [19]. The dataset includes 162 symptoms collected from, as of the time of this updated work, more than 6000 respondents, as well as PEM severity and demographics, offering a uniquely high-dimensional view of symptom burden.

Here, we use this patient-led dataset to examine the robustness of symptom-based Long COVID phenotypes across three distinct clustering approaches: (A) a genetically optimized autoencoder followed by hierarchical clustering (HDBSCAN), (B) an ensemble consensus approach built from multiple UMAP + *k*-means clusterings, and (C) latent class analysis (LCA). Rather than proposing a single best clustering, our goal is to evaluate how strongly cluster assignments depend on algorithm choice and which patient features are reproducibly captured as symptom phenotypes across methods. These answers enable us to explore the implications for clinical practice, trial design, and integration of symptom data with emerging biomarkers.

We hypothesized that any robust structure in this high-dimensional symptom dataset should be detectable and reasonably consistent across different clustering methodologies, whereas non-reproducible structures likely reflect artifacts of a particular method. As such, our multi-method approach aimed to address a fundamental challenge inherent to all unsupervised machine learning, namely the lack of ground-truth labels. Our findings indicate that while each method yields clinically interpretable clusters, cross-method reproducibility is low, and the overall symptom space appears continuous rather than cleanly separable into stable phenotypes. This result underscores the challenges in clustering symptom data and highlights the pivotal role of algorithm selection in shaping outcomes.

## Methods

### Survey

We conducted a cross-sectional analysis of data from an ongoing international survey designed and led by Long COVID patients through the Patient-Led Research Collaborative. Survey design, piloting, and recruitment have been described previously [19, 32]. All but one of the authors are people with Long COVID, and patient researchers were involved in all stages of the project, including survey design, data collection, analysis, and interpretation. The study was approved by the University College London (UCL) Research Ethics Committee [16,159.002] (London, UK), and all participants provided written informed consent with no financial incentives or compensation.

Recruitment began in September 2020. For this analysis, we included responses collected through August 2023. Inclusion criteria were: (i) age ≥ 18 years; (ii) probable or confirmed SARS-CoV-2 infection based on World Health Organization consensus criteria, recognizing that access to testing has been unequal and that Long COVID clinical manifestations do not differ substantially between test-confirmed and probable cases [19]; and (iii) ≥90 days of ongoing symptoms to define Long COVID.

Out of the 14 169 responses we received, the following responses were removed from the dataset: did not start survey (only completed consent form) or did not complete symptom questions (*n* = 5557); flagged as spam by Qualtrics (*n* = 8); symptom onset date before December 2019 (*n* = 71); 0 days of symptoms (*n* = 7); test entry (*n* = 1); duplicated participants (*n* = 247); responses with ongoing symptoms who had not reached 3 months (*n* = 2091); and those who had recovered (*n* = 157). Our final analytic sample was 6031 individual survey responses.

### Symptom assessment

The survey assessed 162 symptoms across 10 domains (e.g. respiratory, cardiovascular, neurological, gastrointestinal). Symptoms were queried at 10 recalled time points: weekly for the first four weeks after symptom onset and monthly up to seven months. For the present clustering analysis, we collapsed across time and coded each symptom as a binary variable indicating whether the respondent reported experiencing that symptom at any surveyed timepoint. This choice prioritizes breadth of symptom coverage and minimizes bias from missing or irregular time series, at the cost of losing detailed trajectory and severity information. Symptoms were grouped into categories (e.g. sleep, speech/language, autonomic, sensory) for visualization and interpretation (Supplementary Data 1, available as supplementary material  *OXFIMM* Journal online). The full text of the survey is available on Figshare [33].

### Post-Exertional Malaise (PEM)

PEM was assessed via direct self-report, reflecting its clinical relevance as a symptom strongly associated with functional impairment and disability in Long COVID and related conditions. Participants were asked whether they experienced worsening or relapse of symptoms following physical and cognitive activity and, if so, to rate the average score of physical and cognitive PEM severity separately on 0–10 scales, where higher scores indicated more severe symptom exacerbation. The survey question specifically referenced symptom fluctuation; however, it did not systematically assess features such as delay of onset, duration of exacerbation, or disproportionality of response. As such, self-reported PEM may encompass heterogeneous experiences of exertion intolerance, not all of which meets the formal criteria for PEM.

The binary presence/absence of PEM question was used as input for the clustering algorithms, but the 0–10 scaled scores were not. The severity scores instead served as external variables to aid interpretation of symptom-based clusters and to assess whether cluster structure aligned with clinically salient dimensions of illness severity. Clustering was performed exclusively on binary symptom indicators to enable the use of unsupervised methods designed for high-dimensional binary data. PEM severity scores were rescaled to the interval [0,1] for analysis, with 1 corresponding to a rating of 10. Where severity averages are reported, they are calculated only among participants who provided a non-zero severity rating.

### Demographics and external variables

Participants reported age in categorical ranges (18–29, 30–39, 40–49, 50–59, 60–69, 70–79, 80+); we used midpoints to approximate mean ages, treating 80 + as 80 years. Gender was categorized as Female/Woman, Male/Man, Non-binary/Genderqueer/Gender non-conforming, Prefer not to say, or Other, and sex was operationalized in the survey as a followup question asking if participants gender matched their gender assigned as birth, with yes or no options. As for PEM severity, demographic information was used when analyzing clusters, but was not included as part of the input to the machine learning algorithms.

Data were cleaned in Python version 3.7.1.

### Analysis

We applied three unsupervised clustering methods to the same 6031 × 162 binary symptom matrix:

- Genetically-optimized autoencoder + HDBSCAN
- Ensemble clustering
- Latent class analysis

All analyses were performed in Python. Code for data preprocessing and clustering is publicly available (https://github.com/leothesouthafrican/PLR).

### Method A: autoencoder + HDBSCAN

Method A learned a non-linear low-dimensional representation of the high-dimensional symptom space and clustered patients in that learned embedding. We first performed feature selection by dropping symptoms that were extremely rare or ubiquitous (near-zero or near-one prevalence), or highly correlated with other symptoms as quantified using skewness and pairwise correlation thresholds (Supp Note A, available as supplementary material  *OXFIMM* Journal online). These thresholds were chosen empirically to balance retaining clinically important symptoms with reducing redundancy and noise; sensitivity analyses indicated that more aggressive feature dropping decreased clinical interpretability.

We then trained an autoencoder to compress the remaining symptom features into a two-dimensional latent representation optimized for reconstruction error. Clustering was performed on this embedding using HDBSCAN, a density-based method that labels low-density points as noise [34]. Hyperparameters were optimized using a genetic algorithm with silhouette score in the embedding space as the fitness function [35], described in detail in Supp Note A, available as supplementary material  *OXFIMM* Journal online. The optimal cluster count was determined by selecting for solutions with the highest silhouette score that had fewer than 13 clusters.

We quantified symptom prevalence within each cluster and defined “enrichment” as the difference between cluster-level and cohort-wide prevalence (*δ*). Symptoms with |*δ*| > 0.1 were considered strongly enriched or dis-enriched.

### Method B: ensemble clustering

Method B used an ensemble of UMAP + k-means-based clusterings to generate a consensus solution. After initial experimentation, we chose UMAP for dimensionality reduction followed by k-means for clustering, with random draws from broad parameter ranges to generate candidate clusterings (Supp Note B, available as supplementary material  *OXFIMM* Journal online). From this library, an ensemble of clusterings was selected using a joint criterion balancing individual clustering quality and diversity [36]. A co-association matrix counting how often each pair of patients appeared in the same cluster across the ensemble was normalized and used as input for spectral clustering, yielding the final consensus clustering.

The full ensemble procedure was repeated 10 times with different random seeds, and cluster number was selected based on eigengap heuristics and stability [37]. Symptom enrichment and external variables (PEM, age, gender) were analyzed as for Method A.

### Method C: latent class analysis

Method C used latent class analysis (LCA) implemented in StepMix v2.1.3 to fit finite mixture models to the binary symptom data [38]. We removed symptoms present in > 95% or < 5% of patients to avoid unstable parameter estimates. Models with 2–25 classes were compared using Bayesian information criterion (BIC); both single-run and multi-run averages supported a 13-class solution (Supp Note C, available as supplementary material  *OXFIMM* Journal online).

For each patient, the model produced a probability of membership in each latent class; we assigned patients to the class with the highest probability. Most patients had high certainty assignments, with only 1.7% of patients having no class with probability > 0.5. We repeated the procedure across 10 random seeds and used a consensus clustering based on co-association plus hierarchical clustering as a sensitivity analysis, confirming that most clusters were reasonably stable. We also examined how the optimal number of clusters under LCA changed as we randomly removed symptoms, mimicking studies using shorter symptom lists, described in detail in Supp Note C, available as supplementary material  *OXFIMM* Journal online.

In contrast to methods A and B, we summarized clusters using model-derived symptom probabilities, defining enriched symptoms as those whose inferred probability in a given class exceeded the average probability across other classes by a cluster-specific threshold (Supp Note C, available as supplementary material  *OXFIMM* Journal online).

### Method comparisons

To compare pairs of clusterings (A versus B, A versus C, B versus C) we computed average mutual information using full patient-level cluster assignments [39], a measure with strong performance when comparing clusterings with different numbers or sizes [40].

When computing the adjusted mutual information (AMI) where one clustering only has a partial label assignment, it is necessary to produce a complete assignment by either predicting or imputing the missing labels. This situation was handled as follows for two different sources of partial label assignment:

1. When subsampling of the dataset to evaluate robustness of a given method: patients not included in the sample were not assigned a cluster label. Model-based clustering method C predicted missing labels based on the model. Ensemble-based method B applied k-nearest neighbor imputation to predict the missing labels.
2. When HDBSCAN assigned cluster label −1: such patients are part of the noise cluster and were considered part of the same group for the purpose of AMI calculation. In practice, we found that retaining the −1 label made little difference as the noise cluster was small (<4% of the dataset for method A).

A detailed note on the creation of t-SNE plots for comparative visualization is provided in Supp Note D, available as supplementary material  *OXFIMM* Journal online.

## Results

**Figure 1:**
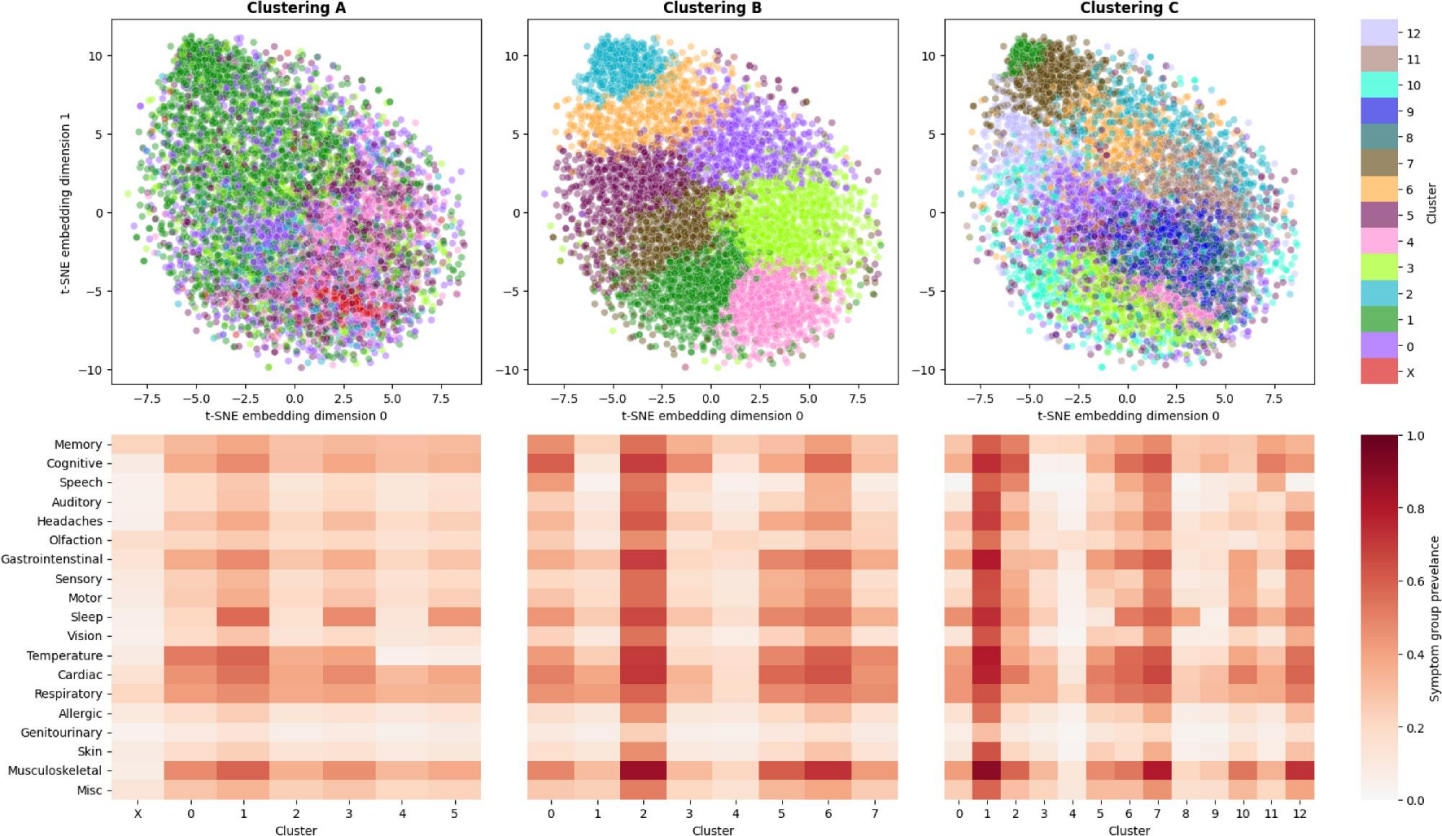
*Left*: Method A (genetically optimized autoencoder) with 6 clusters and one noise cluster; *Middle*: Method B (ensemble) with 8 clusters; *right*: Method C (LCA) with 13 clusters. *Top row*: the 3 clusterings shown in the same 2D t-SNE embedding. Each point represents a patient and each color represents a cluster. Patients in a given cluster are not shared across clusterings, and sharing a cluster number does not imply that two clusters from different methods are similar. *Bottom row*: Average fraction of symptoms reported in each symptom grouping for patients in each cluster. Cluster AX denotes patients who were labeled as noise by HDBSCAN. Symptom groups are defined in Supp Data 1.

The 6031 participants form a self-selected sample and were predominantly middle-aged adults with a median age around the mid-40s and a median illness duration of 198 days (Table 1). Approximately 78% identified as women, consistent with several Long COVID studies reporting higher symptom burden among women, though reported sex distributions vary substantially across studies depending on case definitions, sampling strategies and data sources [41–44]. Based on direct self-report, most participants experienced PEM, with high average levels of physical and cognitive PEM severity, with cohort means of 0.78 and 0.64 on the 0–1 scale respectively. Across all three clustering methodologies, the overall structure of the symptom data appeared relatively continuous. t-SNE embeddings showed gradual gradients in symptom burden and symptom group prevalence rather than distinct clumps corresponding to clearly separable phenotypes (Fig. 1). Similar symptoms tended to co-occur—for example, multiple speech or memory symptoms often occurred in the same patients—but these patterns existed along smooth gradients rather than forming sharply bounded groups (Supp Fig. 1, available as supplementary material  *OXFIMM* Journal online).

**Table 1:** Cohort characteristics summary.

	Categories	Median (IQR)/Fraction
*Age*	18–29, 30–39, 40–49, 50–59, 60–69, 70–79, 80+	44.5 (34.5–54.5)
*Gender*	Woman, Man, Non-binary/Genderqueer/Gender non-conforming	Woman: 0.78, Man: 0.20, Other: 0.02
*Ancestry*	White; Multiple ancestries including white; Asian, South Asian, South East Asian; Hispanic, Latino or Spanish Origin; Other	White: 0.83, Multiple: 0.06, Asian+: 0.3, Hispanic+: 0.3, Other 0.2
*Illness Duration*	Numeric	197 (173.5–254)
*PEM severity*	Physical, Cognitive: numeric. Severity reported only present in 88% of patients who listed PEM as a symptom.	Physical: 8 (7–9); Cognitive 6 (4–8).

### Method A: autoencoder + HDBSCAN

**Figure 2:**
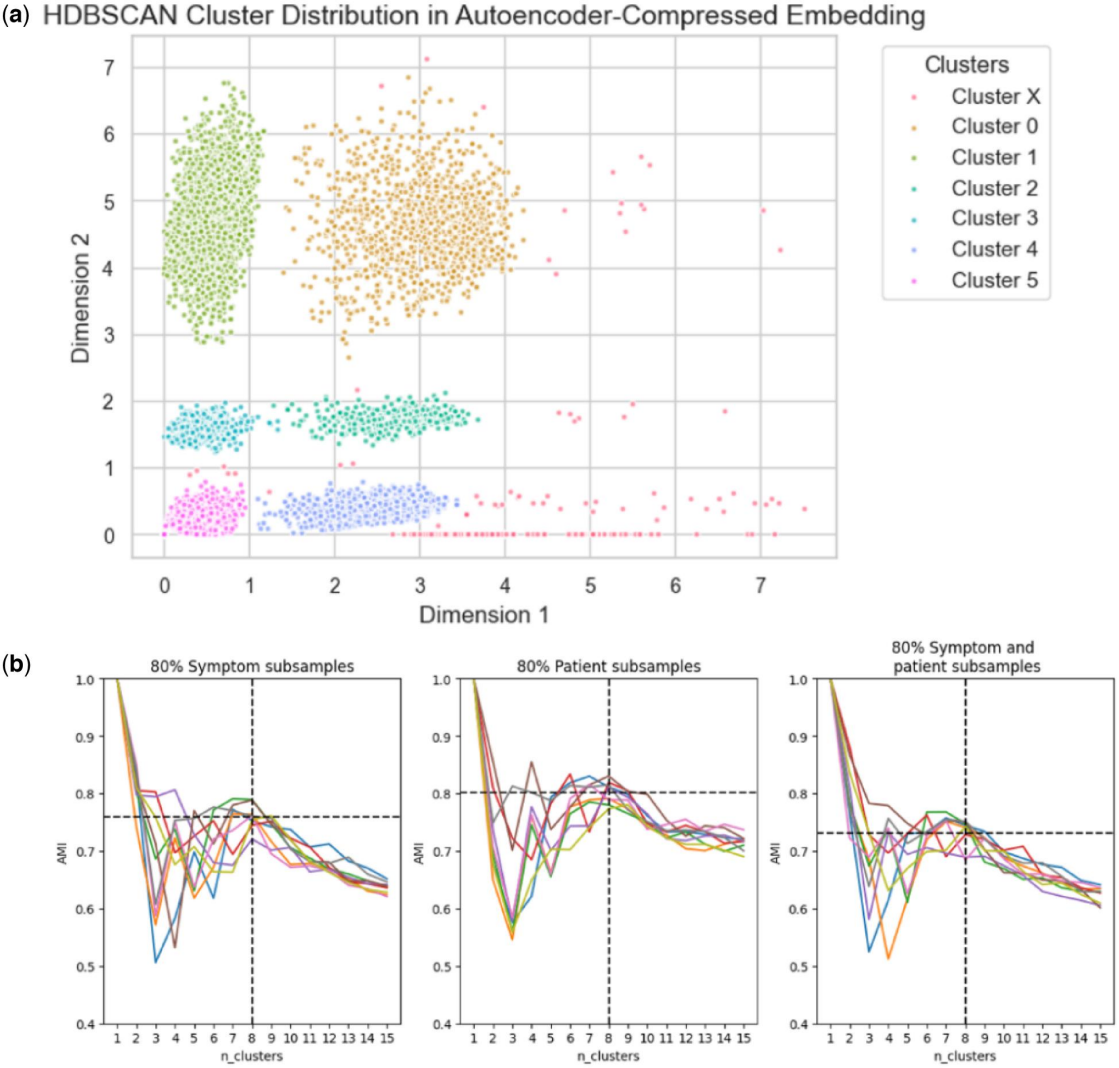
(a) Representation space learned by the method a autoencoder clustered using HDBSCAN. Each point represents a patient, and colors represent the six clusters. The ‘X’ cluster are points labeled as noise by HDBSCAN. The plot dimensions are the compressed representations generated by the autoencoder and capture essential patterns and relationships within the data in a reduced-dimensional space for clustering (Supp Note a). b) Robustness of clustering B under different sub-sampling regimes, as measured by the AMI calculated against the equivalent run of the ensemble method using the full dataset. Each subsampling is repeated 10 times and the black dashed lines show the location of the mean AMI at n_clusters = 8.

Method A identified six main clusters plus a noise/outlier group (AX), with a silhouette score in the autoencoder representation space of 0.6133 (Fig. 2a). Interpretation of the autoencoder dimensions suggested that Dimension 1 primarily reflects sleep disturbance with additional contributions from motor related symptoms, whereas Dimension 2 captures temperature regulation symptoms and, to a lesser extent, gastrointestinal and musculoskeletal related symptoms (Supp Note A: Tables A3 and A4, available as supplementary material  *OXFIMM* Journal online). This indicates that these symptom categories are maximally informative for cluster separation.

The clusters differed meaningfully in overall symptom burden, and, in post hoc analyses, showed differences in self-reported PEM severity and gender composition. Across clusters, we observed a strong positive correlation between the proportion of women and both symptom burden and PEM severity (Supp Fig. 2, available as supplementary material  *OXFIMM* Journal online). Cluster A1, the largest cluster, exhibited the highest overall symptom count and the highest physical and cognitive PEM severity, and had the greatest proportion of women. This group was enriched for sleep disturbance and temperature dysregulation (*δ* = 0.275 and 0.288 with *Z* scores of 1.391 and 1.282, respectively; Supp Data 2, available as supplementary material  *OXFIMM* Journal online).

At the opposite end, Cluster A4 represented a low-burden cluster, with reduced overall symptom count and especially low temperature-related symptoms (mean severity 0.052, *Z*-score −1.0 or −1.2 computed with or without outliers). When comparing Cluster A4 to its nearest-neighbor, cluster A5—which has a symptom burden comparable to the cohort mean—the largest difference (0.313) was in sleep-related symptoms, which were higher in Cluster A4. This suggests that lower sleep disturbance may be a key factor distinguishing patients with lighter overall symptom loads.

The outlier group (Cluster AX) contained patients with the lowest symptom counts, lowest self-reported PEM severity, and a lower proportion of women (Table 2). Their most distinctive feature was especially low cognitive PEM severity, which likely contributed to their classification as noise by HDBSCAN.

**Table 2:** Summary of clustering method A.

Cluster	Name	Size	Symptom count	Women	Age	Physical PEM Severity	Cognitive PEM Severity
*AX*	Outliers	222	16	0.667	47.6	0.619	0.271
*A0*	Temperature dysregulation (chills and flushing sweats). Cardio-respiratory (shortness of breath, heart palpitations and dizziness). Cognition (short term memory and attention/concentration).	1125	44	0.795	47.3	0.773	0.549
*A1*	Largest cluster. Highest proportion of women. Highest symptom burden. Severe insomnia and sleep interruption. Severe physical fatigue and PEM.	**2126**	**60**	**0.830**	46.7	**0.811**	**0.636**
*A2*	Elevated temperature. Generally elevated cardio-respiratory symptoms.	321	35	0.791	45.9	0.747	0.485
*A3*	Very generalized cluster. Symptom severity scattered over: cardio, cognition, short term memory, sleep disturbances, temperature dysregulation and headaches	498	46	0.791	46.3	0.773	0.594
*A4*	Lowest symptom burden of non outlier clusters. Lowest severity of cognitive PEM. Notably low sleep disturbance.	850	28	0.714	**47.7**	0.719	0.480
*A5*	Elevated insomnia, short term memory	889	37	0.721	47.1	0.735	0.530
*Cohort*		6031	42	0.78	47.0	0.778	0.637

For each numeric value the cluster with the highest value is shown in bold. Descriptive names have been assigned to each cluster based on characteristic symptoms, however more details are given in the text. Age corresponds to the midpoint average of the age class of patients within a cluster. Women lists the fraction of patients whose reported gender was woman. Cognitive and physical PEM are averaged reported cognitive and physical symptom severity during PEM respectively, calculated as the average of patients who reported experiencing the reported form of PEM. The largest value in each metadata column is highlighted in **bold**.

Overall, Method A highlights meaningful structures within the symptom data, with sleep and temperature regulation emerging as central drivers of cluster separation, and gender composition aligning tightly with symptom severity patterns.

### Method B: ensemble clustering

This ensemble method yielded an 8 cluster solution with relatively balanced cluster sizes (Table 3, Supp Note B, available as supplementary material  *OXFIMM* Journal online). Clusters clearly divide symptom space when tSNE projected into the embedding used for Fig. 1, with a maximum silhouette score of 0.24, versus −0.03 and 0.01 for clusterings A and C respectively. Clusters were organized along a diagonal axis in the embedding space corresponding to increased symptom burden (Fig. 1, Supp Fig 1, available as supplementary material  *OXFIMM* Journal online). This structure also showed good robustness to the 3 subsampling regimes, with mean adjusted mutual information (AMI) of 0.75, 0.82 and 0.74 with the final clustering B (Fig. 2b).

**Table 3:** Similar to Table 2, but for clustering B.

Cluster	Name	Size	Symptom count	Women	Age	Physical PEM Severity	Cognitive PEM Severity
*B0*	Speech, memory, cognition, sleep and eye/vision (neurological).	615	53	0.833	45.1	0.806	0.739
*B1*	Cognitive PEM much less severe than physical PEM. Shortness of breath, chest burning pain, short term memory, heart palpitations.	872	30	0.748	47.6	0.740	0.478
*B2*	Highest symptom burden. Severe physical and cognitive PEM. Highest proportion of women. Sensory sensitivity, difficulty communicating verbally and processing information, neuropathy, itchy eyes and blurred vision.	506	**89**	**0.866**	45.5	**0.876**	**0.786**
*B3*	Largest cluster. Cognitive dysfunction symptoms most enriched. Cognition, long term memory, speech, PEM, and fatigue.	**1105**	34	0.693	47.3	0.749	0.654
*B4*	Lowest symptom burden. Highest average age. Lowest PEM severity. Altered smell and taste including loss, respiratory.	887	21	0.692	**50.3**	0.676	0.479
*B5*	Nausea, temperature dysregulation, sleep disturbance, paresthesia and vibrations, palpitations, dizziness and vertigo.	727	54	0.834	45.0	0.794	0.611
*B6*	Paresthesia, speech, vision problems, neuropathy, tremors, temperature dysregulation.	611	69	0.856	45.7	0.848	0.736
*B7*	Temperature dysregulation, dry cough, loss of smell, taste and appetite, shortness of breath.	708	40	0.836	47.2	0.763	0.554
*Cohort*		6031	42	0.78	47.0	0.778	0.637

Of note, Cluster B2 had the highest symptom burden, the highest proportion of women, and the highest physical and cognitive PEM severity reported. It showed strong enrichment for sensory sensitivity, speech and communication difficulties, and eye/vision symptoms. In contrast, Cluster B4 had the lowest mean symptom burden (21), highest mean age (50.3), lowest mean cognitive PEM severity (0.48), lowest proportion of women (0.69), and lower prevalence of fatigue and PEM compared to other clusters. Olfactory and taste symptoms were mildly enriched in this group (Supp Note B, available as supplementary material  *OXFIMM* Journal online).

The strong continuity of symptom space gave rise to proximal clusters with significant similarity (Table 3, Supp Note B, Supp Data 3, available as supplementary material  *OXFIMM* Journal online). Clusters B1 and B3 had similar overall symptom burden (30 and 34 respectively) but differed in the balance of cognitive versus physical PEM severity; B3 had higher cognitive PEM severity (0.654) and enrichment of speech and cognitive symptoms, whereas B1 had relatively low cognitive PEM (0.478). B7 was similar to B5 as evidenced by their proximity and overlap in the embedding space. Both clusters shared strongly enriched temperature dysregulation (elevated temperature, chills, flushing, sweats) and respiratory symptoms (*s*hortness of breath, tightness of chest). However, B5 included strongly enriched cognitive and sleep disturbance components which were not present in B7. Conversely, three symptoms were strongly enriched in B7 but dis-enriched in B5: loss of smell (0.51 versus 0.31), loss of taste (0.48 versus 0.28) and fever (0.55 versus 0.37).

### Method C: latent class analysis

LCA identified 13 clusters with varying symptom burden and symptom profiles (Table 4). Each patient was assigned to the cluster which had the highest probability. The output of an individual LCA run is presented as the main output of this method and referred to as clustering C throughout; this result was compared to a consensus clustering defined by combining clusterings with different random seeds (Supp Note C, available as supplementary material  *OXFIMM* Journal online). Symptom enrichment was determined using the inferred likelihood of observing each symptom in a patient from each cluster (Supp Data 4, available as supplementary material  *OXFIMM* Journal online). Only 1.7% of patients had ambiguous assignments, defined as cases where no single cluster had probability above 0.5. Because these patients are a small fraction of the dataset and not overrepresented in any particular cluster, they were assigned to their highest probability cluster for further analysis. The observed clusters have a high degree of coherence among both unusual and prevalent symptoms, with the most distinctive symptoms generally coming from a semantically similar group—for example, the top distinguishing symptoms for cluster C2 were all from the speech symptom subgroup.

**Table 4:** Similar to Table 2, but for clustering C.

Cluster	Name	Size	Symptom Count	Women	Age	Physical PEM Severity	Cognitive PEM Severity
*C0*	Temperature dysregulation, sleep disturbance, cognition (attention/concentration) and shortness of breath	**656**	40	0.794	45.2	0.752	0.595
*C1*	High symptom burden with cognitive dysfunction, difficulty reading and communicating, and sensory and allergic symptoms; Elevated musculoskeletal symptom count; high physical and cognitive PEM severity	178	**101**	0.848	44.0	**0.897**	**0.818**
*C2*	Difficulty reading and communicating, high prevalence of cognitive symptoms	399	56	0.769	45.7	0.804	0.772
*C3*	Low symptom burden, cognitive symptoms reduced with short term memory difficulty still reported	549	27	0.745	47.9	0.701	0.416
*C4*	Lowest symptom burden, Lowest cognitive PEM severity, reduced frequency of PEM, cognitive symptoms reduced	389	11	0.640	47.6	0.637	0.394
*C5*	Chest tightness, dizziness/vertigo, shortness of breath, gasping for air while oxygen normal	352	44	0.835	46.8	0.783	0.593
*C6*	Impaired reading and communication, elevated temperature and chills/sweats, sleep difficulty and elevated average musculoskeletal symptom count	449	58	**0.902**	44.2	0.821	0.703
*C7*	High symptom burden with impaired speech, auditory and visual comprehension, blurred vision, pain: muscle aches, muscle spasms, neuralgia and skin burning without associated rash.	544	78	0.881	45.8	0.854	0.757
*C8*	Insomnia, difficulty falling asleep and waking up in night, lower symptom burden	490	23	0.673	**49.4**	0.732	0.539
*C9*	Consistent sleepers with PEM and cognitive impairment, lower symptom burden	534	24	0.738	47.3	0.696	0.574
*C10*	Insomnia, tremors, sensory features: tingling/prickling, skin burning without rash, vibrations.	509	45	0.800	46.9	0.751	0.555
*C11*	Speech and cognition with moderate symptom burden	608	38	0.745	46.3	0.767	0.682
*C12*	High symptom burden with reduced cognitive/speech, elevated musculoskeletal symptom count	374	63	0.813	45.3	0.820	0.675
*Cohort*		6031	42	0.78	47.0	0.778	0.637

Clusters particularly of note include C1, which had very high symptom burden with enrichment for cognitive dysfunction, communication difficulties, sensory and allergic symptoms, and musculoskeletal pain, combined with the highest severity of both physical and cognitive PEM reported. Cluster C4 had the lowest mean symptom burden (11), lowest cognitive PEM severity, and lower frequency of PEM overall (0.57), though 80% of cluster members still reported short-term memory impairment. Cluster C7 had high symptom burden with prominent speech impairment, auditory and visual symptoms, and pain. Cluster C9 had PEM but had markedly reduced prevalence of self-reported sleep disturbance when compared to the dataset overall: difficulty falling asleep (0.00 versus 0.44), waking up during the night (0.00 versus 0.53), and insomnia (0.03 versus 0.76), indicative of the fact that although PEM is considered a hallmark symptom of ME/CFS, not all Long COVID patients reporting PEM meet ME/CFS diagnostic criteria [21].

We also explored model changes with respect to held out symptoms, focusing on how the optimal number of clusters changes when fewer symptoms are reported. The optimal number of clusters decreased as symptom numbers were reduced. While the full 13 clusters required the full dataset, 70% of symptoms (approximately 100) were required for complexity of 12 clusters, and 10 clusters remained optimal with as few as 30% of the reported symptoms (Supp Fig. 3, available as supplementary material  *OXFIMM* Journal online). This demonstrates that studies with significantly fewer symptoms assessed, may miss clusters otherwise distinguishable.

### Comparative analysis

**Figure 3:**
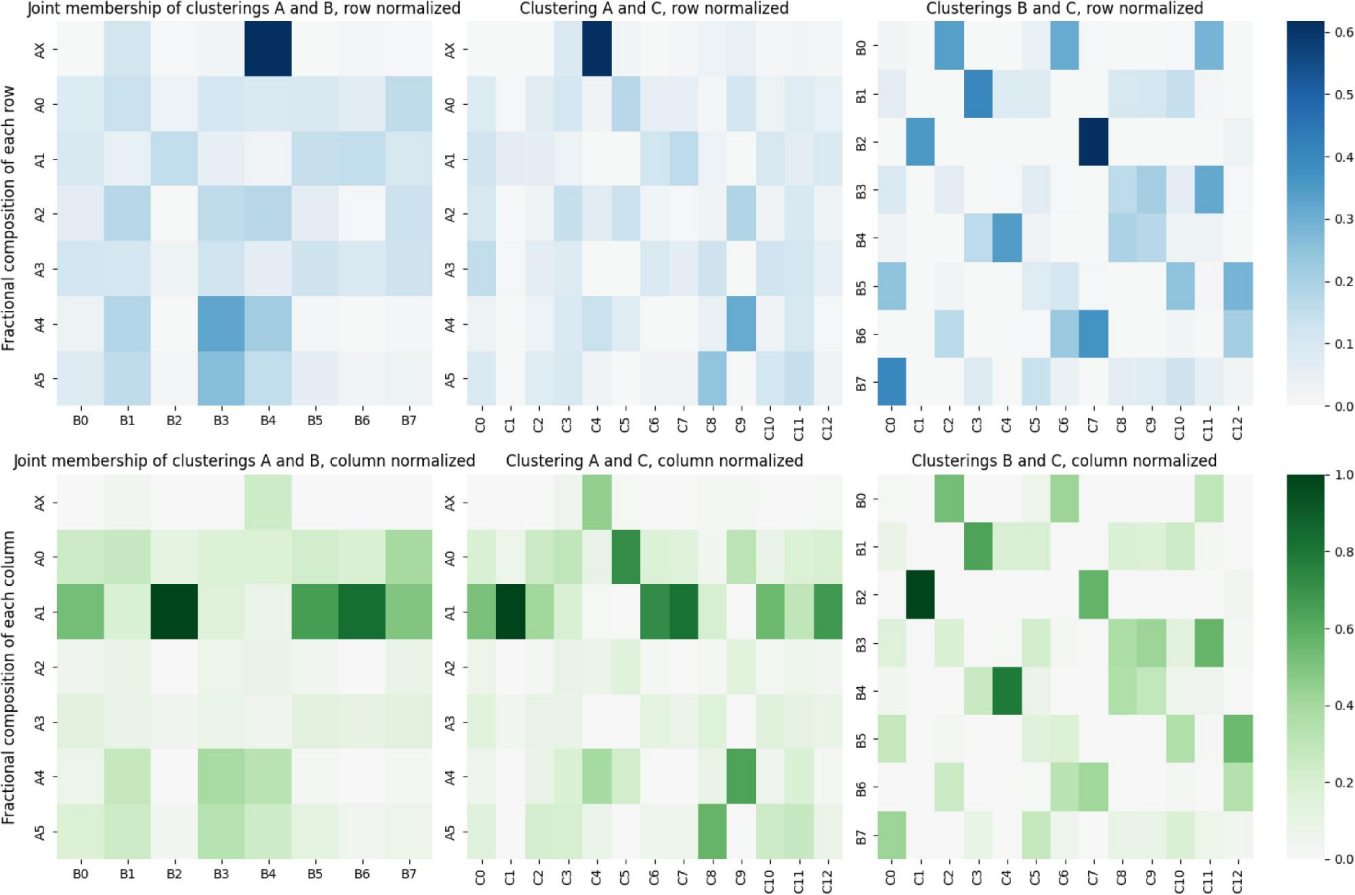
Cluster overlaps between the three methodologies. Top row depicts row-normalized data, in which each square is colored by the fraction of each row which is made up by patients from each column. A dark box indicates that patients from the group corresponding the row are predominantly in the corresponding column. The bottom row of plots is column-normalized, depicting the fraction of each column made up by each row. A dark box indicates that patients from the column cluster are predominantly also assigned to the corresponding row cluster. For example, comparing the joint membership of clusters a and B, cluster B2 is mostly composed of members of cluster A1 (bottom left graph), but cluster A1 is mostly composed of members of B4 (top right graph).

Despite the internal validity of each clustering, cross-method agreement was limited. Pairwise AMIs between methods ranged from 0.13 (A versus B) to 0.40 (B versus C), indicating only modest overlap in patient assignments. Poor separability appeared to be a general feature of the dataset, with manifold learning struggling to produce structured 2D embeddings (Supp Note D, available as supplementary material  *OXFIMM* Journal online). Some recurrent features did emerge, however. A high-symptom-burden, multi-systemic symptom cluster was identified in all three methods (A1, B2, C1), but membership overlapped only partially: most patients in B2 and C1 were contained within A1 81% and 90% respectively), but fewer than 10% of patients in A1 are assigned to B2 or C1 in their respective clusterings (Fig. 3). Low symptom burden clusters B4 and C4 strongly overlapped (C4 making up 34% of B4 and B4 making up 79% of C4) and shared higher average age and lower proportion of women; however, method A assigned many of these patients to the noise cluster AX. This is likely due to the pre-autoencoder dimensionality reduction, in which symptom features were dropped using skewness and correlation measures. Clusters B2 and C7 overlapped substantially, with 306 patients assigned to both clusters, and shared enrichment for speech and communication difficulties, suggesting a reproducible subgroup with prominent cognitive-linguistic impairment, as has been observed elsewhere [45, 46]. Cluster C9 (PEM with minimal self-reported sleep disturbance) shared 304 patients with Cluster A4 another, low-symptom-burden cluster with relatively lower cognitive PEM severity reported despite persistent PEM presence. This patient subgroup exhibiting PEM, a hallmark symptom of ME/CFS, while also not reporting the sleep disturbance required to meet diagnostic criteria, has also been observed elsewhere [21].

Across methods, clusters with lower symptom burden consistently had the highest average age and the lowest proportion of women, whereas high-burden clusters contained higher proportions of women and patients with more severe physical and cognitive PEM. This aligns with other studies reporting higher symptom complexity and burden among women with Long COVID [47, 48]. Physical PEM was more severe than cognitive PEM for 89% of patients, but several clusters identified by Method B (e.g. B0, B3) exhibited relatively more severe cognitive PEM and enrichment of cognitive and speech symptoms, suggesting a subgroup with disproportionate cognitive exertion intolerance.

## Discussion

Using data from a large, patient-led survey of 162 symptoms in 6031 adults with Long COVID, we applied three distinct machine learning clustering methods and found that each method produced clinically plausible symptom clusters, including high- and low-burden groupings and recognizable patterns such as neurocognitive, autonomic, and pain-dominant symptom profiles. However, cluster membership was highly sensitive to algorithm choice: pairwise AMI between methods was low, and even conceptually similar clusters (e.g. high-burden groups) contained different sets of patients depending on the method used. The overall symptom space appeared approximately continuous rather than partitioned into distinct, naturally occurring clusters, making it difficult for any single algorithm to identify robust, reproducible phenotypes.

Some features, however, were reproducible across methods, including the association of high symptom burden and more severe self-reported PEM with a higher proportion of women, aligning with several prior studies demonstrating that female patients with Long COVID had higher symptom frequencies than male patients, and were more likely to have complex multisystemic symptoms [47, 48]. It is also worth noting that not all studies have observed the same proportion, with reported sex distributions varying by case definition, sampling strategy, and data source [41–44]. Differences may also reflect question phrasing; PEM in this study was assessed via self-report of symptom exacerbation following physical or cognitive activity, without requiring delayed onset or disproportionate response, and may therefore capture a broader range of exertion-related symptom exacerbation. Similarly, a subset of patients with prominent speech and cognitive-linguistic difficulties emerged across multiple methods (B0/B3 and C1), and a group reporting PEM without sleep disturbance (A4 and C9). PEM is a hallmark symptom of ME/CFS, which includes fatigue as well as exacerbation of symptoms related to pain, immune dysfunction, neurocognition, gastrointestinal function and sleep disturbance [49–51], including sleep onset latency, frequent awakenings, and apnea, with unrefreshing sleep part of the diagnostic criteria for the illness [52–54]. Unrefreshing sleep and sleep issues that usually require a medical diagnosis (e.g. restless leg syndrome and sleep apnea) were not among the symptoms surveyed in our dataset. Moreover, sleep disorders are multifactorial in nature and can have diverse presentations not necessarily linked to ME/CFS and may occur independent of exertion intolerance [55, 56]. Additionally, given the limitations of our measurement, we cannot determine definitively whether the symptom exacerbation captured here reflects PEM as defined in ME/CFS, or represents a broader category of exertion intolerance. Thus, we cannot with complete certainty state that patients in this cluster experience PEM without meeting ME/CFS diagnostic criteria; however, such a population has been observed elsewhere [21, 51], and the reproducibility of the cluster in our dataset suggests that this patient subgroup exists in this cohort as well.

In totality, our results indicate that symptom-based phenotypes of Long COVID are not uniquely defined by the data, instead depending strongly on modeling decisions. In a setting where clusters are being used to design clinical trials and interpret biomarker findings, this instability has significant implications. These findings differ from those of several phenotyping studies that have reported relatively stable symptom clusters using fewer symptoms or different data sources. For example, cluster analyses in post-hospital cohorts have identified respiratory-dominant, neurocognitive, and fatigue/pain clusters that appear reproducible within their datasets [41, 42]. Studies based on outpatient cohorts and EHR data have likewise identified subgroups characterized by neurocognitive symptoms, autonomic dysfunction, or multi-system involvement [28, 57].

In contrast to many other Long COVID phenotyping studies, our sample reflects a self-selected, non-random cohort of often severely impacted community members rather than patients gathered through their interaction with the medical system, and includes many participants with PEM and multi-system symptoms that may be missed in EHR-only cohorts, which lack robust discernment of PEM presence due to the lack of a PEM ICD-10 code [58]. PEM in this study was identified via self-report via survey questions and severity ratings rather than through biomedical or clinical assessment, which may contribute to misclassification and impacts generalizability. We included 162 symptoms, while most prior clustering studies assessed 12–40 [24, 59, 60]. Sensitivity analysis for the LCA clustering method, which mathematically optimizes cluster count using BIC, showed that the optimal number of clusters decreased markedly as symptoms were removed, suggesting that shorter symptom lists may miss important heterogeneity and impose smaller, more stable but potentially less-representative, cluster structures. Additionally, previous studies have not directly compared different clustering algorithms on the same symptom dataset, preventing the method robustness analysis available via direct comparison here. Our findings demonstrate the dependence of clusters on symptom selection, sampling frame, and analytic approach, and complement calls to integrate symptom phenotypes with immunologic and biomarker data to define biologically meaningful endotypes, rather than relying solely on symptom cluster boundaries to define patient subgroups [61, 62]. Additional discussion of how implementation of the algorithms we used could be enhanced is provided in Supp Note D, available as supplementary material  *OXFIMM* Journal online.

Limitations of this study include that it does not include children and adolescents, does not account for asymptomatic organ damage, and reflects a cohort with a median illness duration of 198 days at time of survey. As a questionnaire-based, patient-led study recruited primarily through online channels, the sample is self-selected and may preferentially capture individuals with higher symptom burden, greater functional impairment, or symptoms that are poorly recognized or managed in clinical settings. Accordingly, some of our observed symptom frequencies indicate that our cohort may be non-representative Long COVID populations observed in other studies. In particular, PEM is identified only in a subset of Long COVID studies, with prevalence varying widely depending on study design, symptom definitions, sampling frame, and whether PEM is explicitly assessed [21–23, 63]. While a recent meta-analysis estimated PEM prevalence at approximately half of participants across studies, this estimate is drawn from substantial heterogeneity across studies and should not be interpreted as a universal feature of Long COVID [23]. In our cohort, 88.4% of our participants reported experiencing PEM, which may reflect the patient-led nature of recruitment, explicit querying of PEM and differences in question wording, which has been shown to significantly influence rates of PEM reported [64]. In particular, in this study, PEM was defined for survey takers as worsening or relapse of symptoms following physical or cognitive exertion, likely capturing more individuals than questions also requiring delayed onset of symptom exacerbation. Prior work has found that most Long COVID patients who do not meet ME/CFS diagnostic criteria nonetheless exhibit exertion intolerance with shorter-duration PEM < 14 hours [63]; some of the difference between our survey and other results may be patients with this form of exertion intolerance, as duration of exacerbation was not included in our analysis. It is also possible that patients with Long COVID that maps onto known diagnoses, such as those who develop post-COVID diabetes, are less likely to participate in Long COVID research studies, as their illness is better understood biomedically [65]. Such dynamics may impact many aspects of the cohort composition, including observed sex distributions, and limit generalizability to the broader post-COVID population.

An additional limitation is that the survey did not systematically capture reinfection status at the time this analysis was conducted. Some respondents may have experienced multiple SARS-CoV-2 infections, but we were unable to distinguish initial infection from reinfection in this dataset. As discussed above, on the analysis side, feature-dropping thresholds, dimensionality reduction strategies, and algorithmic assumptions differed across methods, each of which may influence cluster structure and contribute to non-reproducibility. In particular, the methods used here vary in their sensitivity to noise, assumptions about cluster separability and homogeneity, and stability with respect to initialization and parameterization. However, even when preprocessing was aligned to the extent feasible, the core findings of low cross-method agreement and continuous symptom structure remained. A detailed discussion of method-specific limitations and potential avenues for improving stability is provided in Supplementary Note D, available as supplementary material  *OXFIMM* Journal online.

Visualizations suggested that symptom burden and symptom group prevalence vary smoothly, with no obvious natural breaks (Supp Fig. 1, available as supplementary material  *OXFIMM* Journal online). In such a landscape, any clustering algorithm will partition the space, but the exact boundaries will depend on modeling assumptions, leading to algorithm specific phenotypes as observed here. Long COVID likely reflects multiple overlapping mechanisms (e.g. microvascular dysfunction, autoimmunity, persistent viral reservoirs, reactivation of latent infections) [2, 66]. Patients can plausibly exhibit features of multiple endotypes simultaneously, making single-label symptom clusters an imperfect representation. Additionally, the survey data used does not include information as to symptom severity, intensity, or duration, which can vary substantially in Long COVID [19, 67]. The incapacity to distinguish symptom severity and duration may blur clinically important distinctions. Instruments such as the DePaul Questionnaire for PEM [68] and the symptom burden questionnaire for Long COVID [69] may better capture gradations in symptom severity and impact. Despite these challenges, all three methods employed in this work are designed to tolerate noise and recover structure where it exists: Method A by explicitly including a noise structure, Method B by averaging over many base clusterings, and Method C by modeling residual variation probabilistically. Their collective failure to identify shared, discrete phenotypes suggests that the symptom data, as measured here, does not naturally segregate into a small set of stable phenotypes.

For clinicians, our findings argue for caution in treating symptom-based Long COVID clusters as distinct diagnostic entities. An individual patient might be assigned to a high-burden neurocognitive cluster by one algorithm and a more generalized multi-system cluster by another, with no clear way to determine which is ‘correct’, and it is important to remember that symptom clusters are not ‘types’ of patients. Recurrent patterns are still useful, however. Women were over-represented in high-burden clusters and under-represented in low-burden clusters; high symptom burden was associated with more severe PEM; and subgroups with prominent speech and cognitive-linguistic impairment appeared across all methods, consistent with emerging literature on language and cognitive deficits in Long COVID [45] and pointing to a need for structured evaluation of PEM and cognitive-linguistic function. Moreover, we demonstrate that PEM, as captured by this dataset, is not monolithic. Differences in the ratio of cognitive to physical PEM across subgroups, and the robust observation of patients with PEM but minimal self-reported sleep disturbance, suggests multiple PEM-related phenotypes, aligning with work in ME/CFS and Long COVID indicating diverse PEM triggers and trajectories [29, 70] and suggesting a need to probe physical and cognitive triggers separately and focus on individualized pacing and rehabilitation strategies. More broadly, our results support using symptom clusters as exploratory tools to generate hypotheses and help communicate about patterns of illness, rather than as rigid labels that define eligibility or predict response to specific treatments. With any clustering, caution is needed to ensure that algorithmically imposed boundaries are not mistakenly interpreted as biologically discrete or clinically stable subtypes, particularly in conditions such as Long COVID where symptom burden varies along largely continuous dimensions and cluster membership is highly sensitive to analytic choices.

Our multi-method analysis highlights several methodological lessons for future Long COVID phenotyping. Taken together, our findings suggest that clustering studies of Long COVID patients need to be skeptical of cluster outputs, and particularly cluster reproducibility. Machine learning algorithms excel at finding statistically meaningful data subdivisions, but in the context of a homogeneously varying dataset, these partitions are fundamentally arbitrary. Studies should report not only internal validity metrics (e.g. silhouette scores) but also sensitivity of clusters to algorithm choice, parameter settings, and subsampling of patients and symptoms, particularly in cases where analysis of the data to be clustered suggests a continuous symptom space. Incorporating symptom severity, trajectory, and biological markers are all likely to be helpful in defining endotypes, and future work should focus on whether clusters defined jointly in symptom-biomarker space are more robust and clinically useful. While studies with fewer reported symptoms may achieve more consistent results, these outcomes may be artifacts of the limited number of symptoms under consideration. More research is needed to determine not only what symptoms, but what conditions and biological markers can help consistently reproduce Long COVID phenotypes.

## Supplementary Material

iqag010_Supplementary_Data

## Data Availability

Data available upon reasonable request to the corresponding author. Code for data preprocessing and clustering is publicly available (https://github.com/leothesouthafrican/PLR).
