# Supplementary material for: Algorithm dependence of patient phenotypes in Long COVID: a patient-led, multi-method clustering of 6031 patients using 162 self-reported symptoms
Source: Oxf Open Immunol. 2026 Jun 13;7(1):iqag010. doi: 10.1093/oxfimm/iqag010 (PMC13284999; doi:10.1093/oxfimm/iqag010)
Supplement: iqag010_Supplementary_Data [file iqag010_supplementary_data.zip › Supplemental_Figures.pdf]

Supplemental Figures

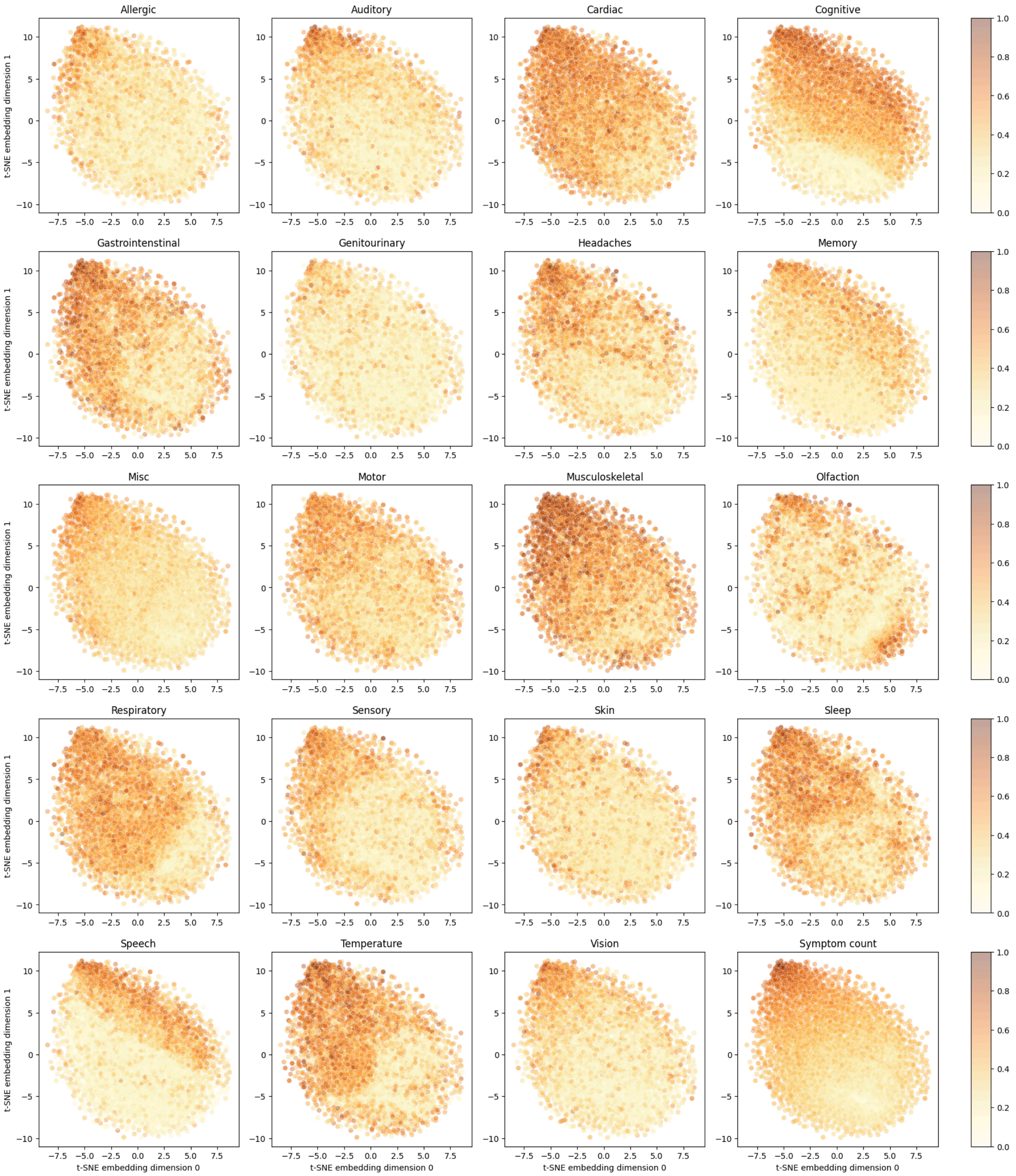

Supplemental Figure 1: The mean values for the symptom groups across the dataset, plotted using the same t-SNE embedding as for Fig 1 in the main text. Symptoms in each group are defined in Supp Table 1.

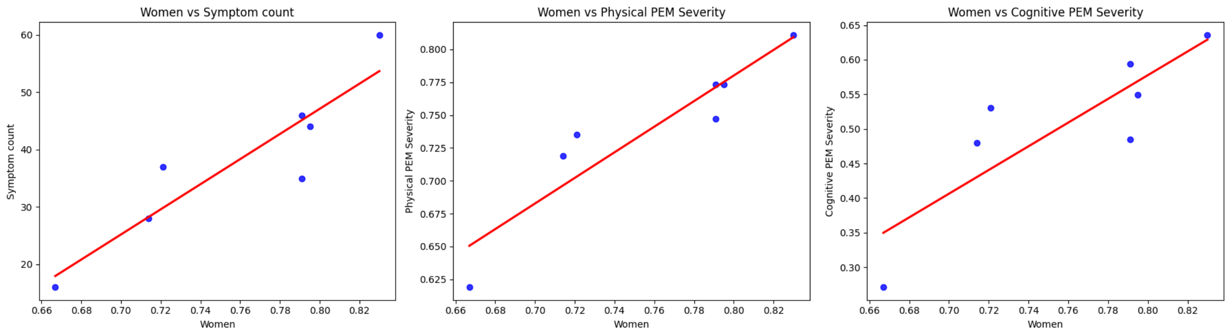

Supplemental Figure 2: Correlations between demographic variable “Women” and outcomes across clusters in Method A. We found a positive correlation between the fraction of women in a cluster and symptom count: (r=0.883, p=0.008, df=6), Physical (r=0.973, p=0.0002, df=6) and Cognitive PEM severity (r=0.883, p=0.008, df=6).

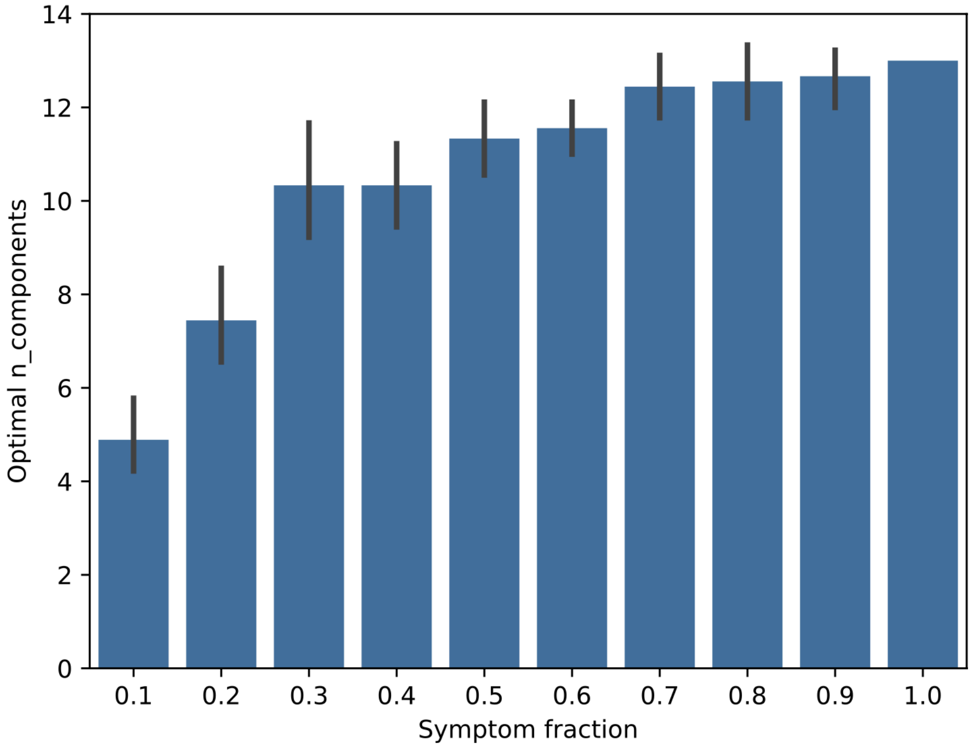

Supplemental Figure 3: Optimal StepMix n_components as determined by BIC for decreasing fractions of measured symptoms.
