# Supplementary material for: Algorithm dependence of patient phenotypes in Long COVID: a patient-led, multi-method clustering of 6031 patients using 162 self-reported symptoms
Source: Oxf Open Immunol. 2026 Jun 13;7(1):iqag010. doi: 10.1093/oxfimm/iqag010 (PMC13284999; doi:10.1093/oxfimm/iqag010)
Supplement: iqag010_Supplementary_Data [file iqag010_supplementary_data.zip › Supplementary_Notes.pdf]

Supplementary Note A: Supplemental information for method A.

We developed a neural network based approach to cluster the high-dimensional symptom data. We first refined the original feature set by dropping some symptom features, which was achieved through setting two thresholds: one based on dropping features skewed heavily towards either 0 or 1 and the other based on symptom correlation (Figures A1 and A2, Table A1), effectively allowing us to reduce the dimensionality of the dataset. This approach was inspired by the questionnaire being launched in the early stage of the pandemic where relevant symptom features were still not clear.

Since clustering techniques perform better in lower dimensions (Domeniconi *et al.* 2007), we subsequently employed an autoencoder neural network to learn a 2-dimensional embedding of the dataset. HDBSCAN (Campello, Moulavi and Sander 2013) clustering was then applied to the resulting dataset. Hyperparameters, including the skewness and correlation thresholds, the number of layers and neurons per layer, and finally, the HDBSCAN parameters were all optimized using a genetic algorithm that utilized the silhouette score (Rousseeuw 1987) as a measure of individual fitness (Table A2). We further refined our approach by using the genetic algorithm's outcomes to instead define a narrower space for a grid search. The outcomes of this secondary optimisation had greater clinical interpretability. For robustness, an autoencoder pipeline, initialized with Glorot initialization (Glorot and Bengio 2010), was run five times with different initial weights and the AMI was calculated across clusters.

To enhance the interpretability of Main Text Figure 2a, the embedded meaning of both of the dimensions was estimated by taking as input a set of clusters C = {1, 2, ..., N}, a set of cluster pairs P = {(i, j) | i, j ∈ C}, and a set of features F = {1, 2, ..., M}. For each cluster pair (i, j) ∈ P, the algorithm calculates the absolute difference d_ijk = |x_ik - x_jk| for each feature k ∈ F, where x_ik and x_jk are the values of feature k in clusters i and j, respectively. It then selects the top 3 features T_ij ⊆ F with the greatest distances d_ijk and stores the results as tuples (i, j, k, d_ijk) in a set R. Finally, the algorithm sorts the elements of R based on the cluster pairs (i, j) and distances d_ijk in descending order and outputs the top 3 feature differences for each cluster pair. The results indicate that Dimension 1 most likely pertains primarily to sleep disturbances but also to motor related symptoms (Table A3) and that Dimension 2 relates to temperature regulation symptoms and to a lesser extent gastrointestinal and musculoskeletal related symptoms (Table A4).

In the analysis of our clusters, we used symptom prevalence as a key measure. This is defined as the mean of the binary symptom value over a group of patients and is equivalent to the frequency of occurrence of the symptom within that group. To understand which symptoms were characteristic of each cluster we defined the metric δ as the difference between the symptom prevalence at the cohort level and the prevalence within the cluster. Symptoms with $\delta>0$ and $\delta<0$ are referred to as enriched and dis-enriched respectively. Those symptoms with $|\delta|>0.1$ are considered to be strongly (dis-)enriched, whereas $|\delta|<0.1$ is referred to as mild. These enriched and dis-enriched symptoms were used to define cluster characteristics for ease of interpretation. The listed characteristics sometimes combine multiple symptoms. They were chosen for

interpretability and should not be taken as fully descriptive. Complete symptom prevalences and enrichments for Method A available in Supp Data 2. A Ward method dendrogram was created to further demonstrate similarity between clusters (Figure A3).

References

Campello RJGB, Moulavi D, Sander J. Density-Based Clustering Based on Hierarchical Density Estimates. In: Pei J, Tseng VS, Cao L, et al. (eds.), *Advances in Knowledge Discovery and Data Mining*. Berlin, Heidelberg: Springer, 2013, 160–72.

Domeniconi C, Gunopulos D, Ma S *et al.* Locally adaptive metrics for clustering high dimensional data. *Data Min Knowl Discov* 2007;**14**:63–97.

Glorot X, Bengio Y. Understanding the difficulty of training deep feedforward neural networks. *Proceedings of the Thirteenth International Conference on Artificial Intelligence and Statistics*. JMLR Workshop and Conference Proceedings, 2010, 249–56.

Rousseeuw PJ. Silhouettes: A graphical aid to the interpretation and validation of cluster analysis. *J Comput Appl Math* 1987;**20**:53–65.

Tables

Approach	Optimised Threshold	Features
Skewness	0.2 < X <0.8	Symptom_Eye_Vision_loss_of_vision: 0.0111, Symptom_Reproductive_early_menopause: 0.0139, Symptom_Reproductive_postmenopausal_bleeding: 0.0161, Symptom_Hallucinations_other: 0.0234, Symptom_SkinAllergy_shingles: 0.0257, Symptom_Heightened_taste: 0.0308, Symptom_Sensations_facial_paralysis: 0.0320, Symptom_Hallucinations_tactile: 0.0337, Symptom_Reproductive_testicle_penis_pain_changes: 0.0365, Symptom_Eye_Vision_tunnel_vision: 0.0383, Symptom_New_anaphylaxis: 0.0429, Symptom_Speech_other: 0.0431, Symptom_Coughing_up_blood: 0.0479, Symptom_Ear_numbness: 0.0494, Symptom_Inability_to_yawn: 0.0541, Symptom_Speech_changes_to_secondary_languages: 0.0557, Symptom_SkinAllergy_other_itchy: 0.0574, Symptom_Sensations_facial_pressure_other: 0.0602, Symptom_Sensations_facial_pressure_right: 0.0680, Symptom_Hallucinations_auditory: 0.0711, Symptom_Sensations_facial_pressure_left: 0.0793, Symptom_Eye_Vision_double_vision: 0.0796, Symptom_Dermatographia: 0.0804, Symptom_Heightened_smell: 0.0822, Symptom_Eye_Vision_redness_outside_eye: 0.0832, Symptom_Respiratory_other: 0.0861, Symptom_Cognitive_Functioning_other: 0.0871, Symptom_Memory_no_new_memories: 0.0875, Symptom_Reproductive_other_menstrual_issues: 0.0900, Symptom_Speech_speaking_unrecognizable_words: 0.0947, Symptom_Skin_Allergy_other: 0.0947, Symptom_Reproductive_abnormally_heavy_periods_clotting: 0.0953, Symptom_Hearing_loss: 0.0970, Symptom_Phantom_taste: 0.0990, Symptom_Hallucinations_visual: 0.1033, Symptom_Cognitive_Functioning_agnosia: 0.1083, Symptom_SkinAllergy_brittle_nail: 0.1094, Symptom_New_allergies: 0.1111, Symptom_Reproductive_sexual_dysfunction: 0.1129, Symptom_Memory_other: 0.1200, Symptom_Headaches_brain_warmth: 0.1205, Symptom_Reproductive_abnormally_irregular_periods: 0.1207, Symptom_SkinAllergy_heighted_reaction_allergies: 0.1239, Symptom_Peeling_skin: 0.1260, Symptom_Sleep_apnea: 0.1262, Symptom_Eye_Vision_pink_eye: 0.1270, Symptom_Fainting: 0.1277, Symptom_Sensations_numbness_one_sided_weakness: 0.1282, Symptom_Covid_toes: 0.1290, Symptom_Headaches_other: 0.1333, Symptom_Eye_Vision_seeing_things_peripheral_vision: 0.1336, Symptom_Memory_forget_tasks: 0.1380, Symptom_Eye_Vision_bloodshot_eyes: 0.1411, Symptom_Vomiting: 0.1499, Symptom_Reproductive_other_urinary_issues: 0.1535, Symptom_Lucid_dreams: 0.1567, Symptom_Bladder_control: 0.1569, Symptom_Petechiae: 0.1632, Symptom_Bradycardia: 0.1643, Symptom_Bulging_veins: 0.1648, Symptom_Slurring_words: 0.1670, Symptom_Speech_difficulty_communicating_writing: 0.1673, Symptom_Cognitive_Functioning_fastthoughts: 0.1733, Symptom_Rattling_breath: 0.1749, Symptom_Reproductive_all_menstrual_issues: 0.1836, Symptom_Restless_leg: 0.1899, Symptom_Acute_confusion: 0.1935, Symptom_Low_temp: 0.1942
Correlation	0.5	Symptom_Loss_of_smell - Symptom_Loss_of_taste: 0.72<br>Symptom_Eye_vision_symptoms - Symptom_Eye_Vision_blurred_vision: 0.68<br>Symptom_Speech_difficulty_finding_words - Symptom_Speech_difficulty_communicating_verbally: 0.65<br>Symptom_Insomnia - Symptom_Waking_up_in_night: 0.64<br>Symptom_Speech_difficulty_communicating_verbally - Symptom_Speech_difficulty_speaking_complete_sentences: 0.58<br>Symptom_Speech_difficulty_finding_words - Symptom_Speech_difficulty_reading_processing_text: 0.58<br>Symptom_Insomnia - Symptom_Difficulty_falling_asleep: 0.55<br>Symptom_Speech_difficulty_finding_words - Symptom_Speech_difficulty_speaking_complete_sentences: 0.55<br>Symptom_Temp_lability - Symptom_Elevated_temp: 0.54<br>Symptom_Altered_taste - Symptom_Altered_smell: 0.53<br>Symptom_Speech_difficulty_communicating_verbally - Symptom_Speech_difficulty_reading_processing_text: 0.52

Table A1: Features dropped before final clustering based on optimized skewness and correlation thresholds. Features that were highly skewed (most patients had a symptom present or no patients had that symptom) were dropped. Features that were heavily correlated with one another were also dropped.

Parameters:

Algorithm	Parameter	Search Space	Optimized Parameter
Autoencoder	Hidden Layer Size	(32, 64, 128, 256)	64
Autoencoder	Latent Dimension	(2, 8, 16, 32, 64)	2
Autoencoder	Learning Rate	(0.001, 0.01)	0.01
Autoencoder	Epochs	(250, 500, 1000)	500
HDBSCAN	Minimum Cluster Size	(20, 50, 100, 125, 150)	135 (refined manually)
HDBSCAN	Minimum Sample Size	(2, 5, 10, 15)	2

Table A2: Parameter search space used for optimisation of autoencoder embeddings and HDBSCAN clustering.

Cluster Pair	Feature	Distance
0-1	**Sleep**	**0.361**
	Motor	0.111
	Musculoskeletal	0.110
2-3	**Sleep**	**0.308**
	Musculoskeletal	0.014
	Headaches	0.095
4-5	**Sleep**	**0.313**
	Motor	0.076
	Cardiac	0.071

Table A3: Dimension 1 interpretation of axes for Fig 2; for each cluster pair above norm distance was calculated across all features. Feature differences between pairs indicate that there is variance in this feature along the axis. These pairs were chosen on the basis that they are similar when compared along Dimension 2 but dissimilar along Dimension 1.

Cluster Pair	Feature	Distance
1-5	**Temperature**	**0.517**
	Muskuloskeletal	0.203
	Gastrointestinal	0.193
0-4	**Temperature**	**0.464**
	Gastrointestinal	0.153
	Muskuloskeletal	0.149

Table A4: Dimension 2 interpretation of axes for Fig 2; for each cluster pair above norm distance was calculated across all features. Features differences between pairs indicate that there is variance in this feature along the axis. These pairs were chosen on the basis that they are similar when compared along Dimension 1 but dissimilar along Dimension 2.

Figures

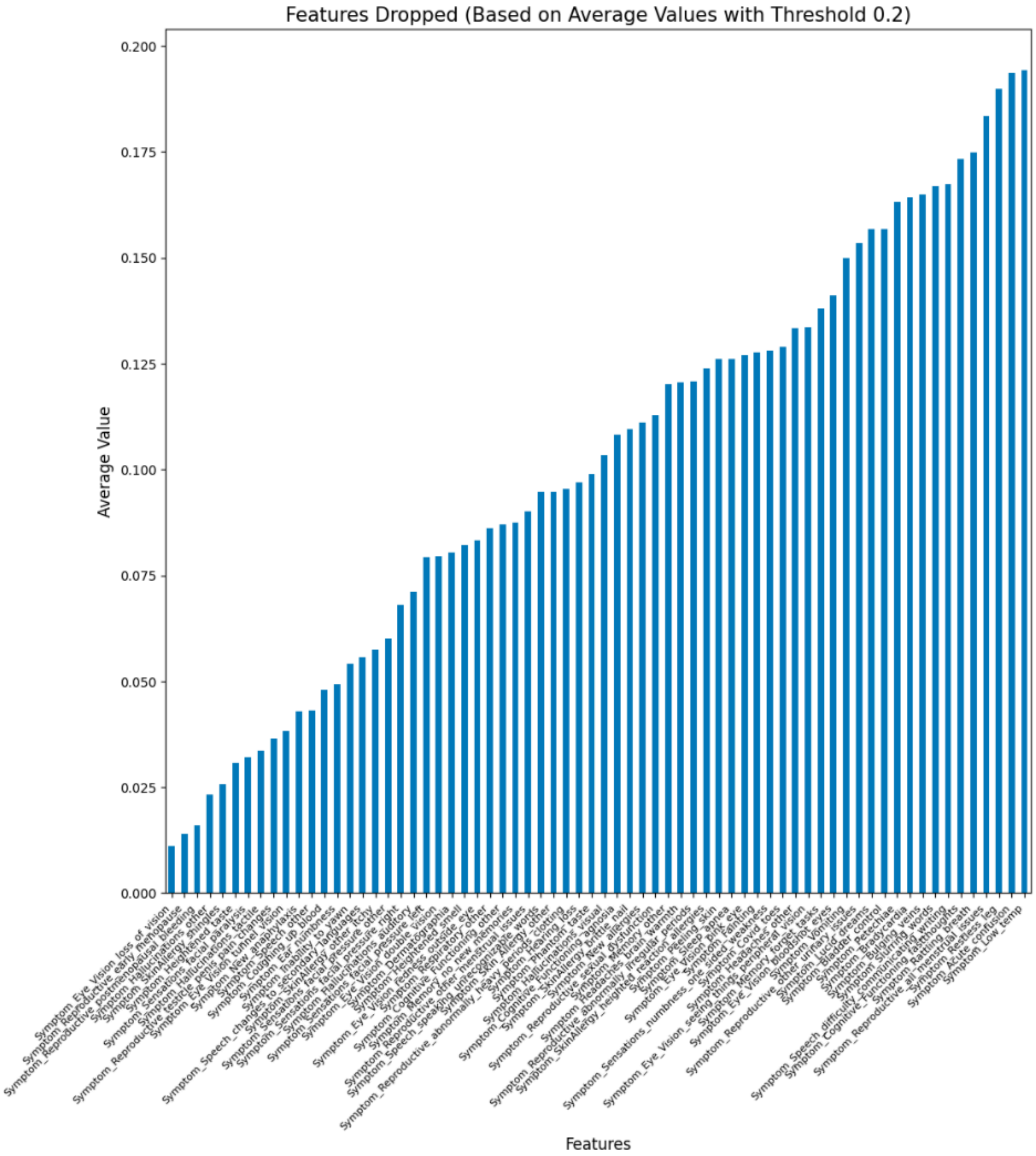

Figure A1: Binary symptom features were excluded based on their average values being either below 0.2 or above 0.8.

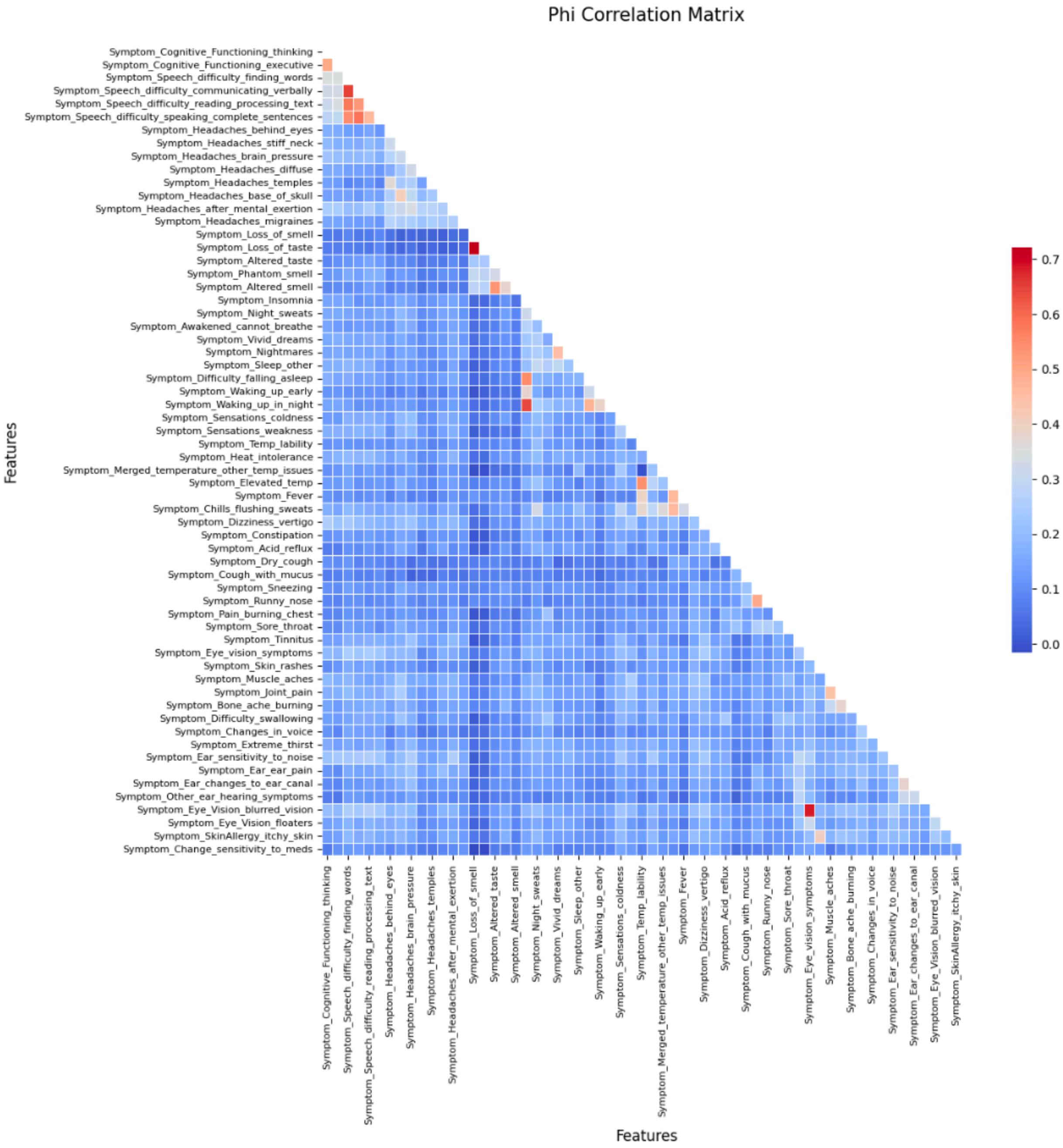

Figure A2: Features remaining after the initial filtration were further analyzed for Phi correlation; those with a correlation exceeding 0.5 were dropped.

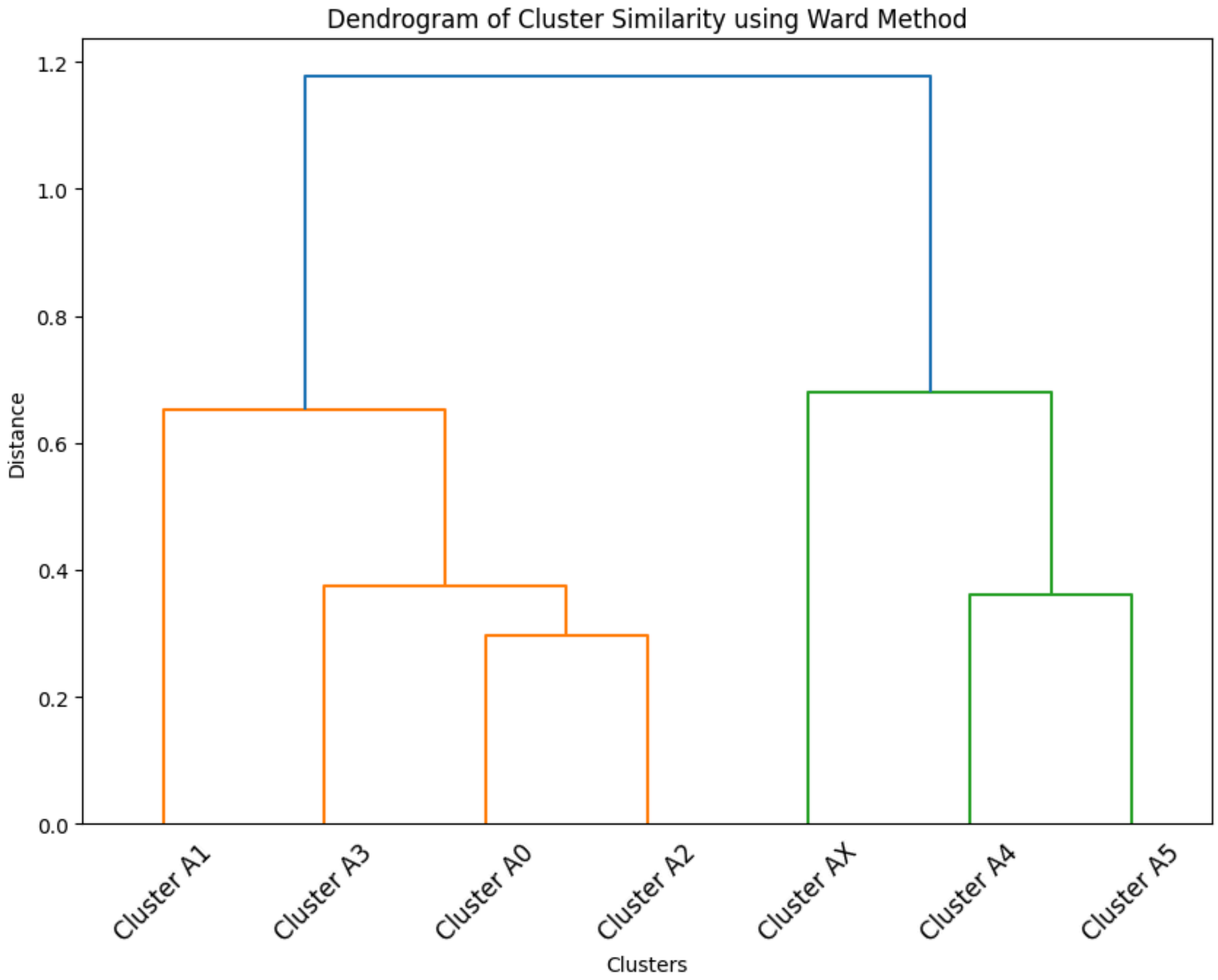

Figure A3: Ward Method dendrogram showing similarity between clusters of solution A.

Supplementary Note B: Supplemental information for Method B

Methods

We developed an ensemble clustering method based on a pipeline consisting of a dimensionality reduction algorithm and a shallow clustering algorithm. We initially experimented with both PCA and UMAP as dimensionality reduction methods, and both k-means and HDBSCAN as clustering algorithms. We ran at least 1000 randomly parameterized clusterings using the 8 combinations of these methods with parameter values drawn from the ranges given in table Table B1.

This stage of the ensemble clustering method is referred to as 'library generation' and the goal is to produce a large and diverse set of high-quality clusterings from which to select the ensemble. One way to do this is to perform multiple runs of the same stochastic algorithm with different random seeds (Boongoen and Iam-On 2018). However, given the complexity of the dataset we felt it would be better to use multiple algorithms with diverse parameterizations. However, HDBSCAN was found to work poorly with this approach and tended to label most patients as noise. For example, for 1000 randomly parameterised runs of UMAP + HDBSCAN, the mean and standard deviation of the fraction of the dataset labeled as noise were 0.75 and 0.08 respectively. This poor performance of HDBSCAN, which persisted under manual parameter exploration, reflects the high level of noise in this dataset and the resulting difficulty in producing robust cluster assignments. We therefore determined to use only k-means as the clustering algorithm. For simplicity we decided to use only UMAP as the dimensionality reduction method, as it is more powerful than PCA with the ability to capture more complex structures from this high dimensional dataset. Therefore our base clusterings were all generated from the single pipeline consisting of UMAP + k-means, and the diversity of our ensemble relied on the random parameterisation of these algorithms using values selected from the ranges given in Table B1.

We employed an ensemble selection method (Fern and Lin 2008) that greedily adds clustering solutions in order to jointly optimize the 'quality' and 'diversity' of the ensemble. We then applied a standard consensus function (Boongoen and Iam-On 2018) to aggregate the ensemble solutions into a single clustering. This consensus function involves building a co-association matrix A that counts how many times each pair of patients occur in the same cluster across the ensemble, normalizing this matrix by the size of the ensemble such that each element $a_{ij} \in (0, 1)$, and then running this matrix through a similarity-based clustering algorithm. In our case, spectral clustering was found to produce the most stable solutions.

The full ensemble method was repeated 10 times with different random seeds to assess the stability of the solution. The optimal number of clusters was chosen using a combination the eigengap heuristic (von Luxburg 2007) and the stability of the clusters. The full ensemble method was repeated using three subsampling regimes to determine robustness to data removal. The three regimes used random samples of 1) 80% of symptoms; 2) 80% of patients; and 3) 80% of both patients and symptoms.

Results

An ensemble of 50 clusterings was selected using the joint criterion (Fern and Lin 2008)from a library of 500 base k-means clusterings, and this ensemble was used to construct a co-association matrix A. Details of the selected ensemble are provided in section SB.2. The eigenvalues of the normalized graph Laplacian L_{norm} invoked by A were computed (Zelnik-Manor and Perona 2004), and the largest eigengaps were used to select candidates for the optimal number of clusters (Fig B1, left panel). The stability of the resulting structures was then assessed by repeat clustering using different random seeds and the most stable was found to be that with 8 clusters with a mean AMI of 0.88 across ten repeats (Fig B1, right panel). It should be noted that 6 clusters was also a strong candidate, with an AMI of 0.85. However, the 8-cluster structure was preferred due to its slightly better stability and the increased clinical discrimination enabled by more groups.

There was a good diversity of base clusterings in the library. Fig B2 shows the clusterings with the lowest and highest AMI values compared to the final clustering B from the main text. The mean AMI value in the library was 0.62, and all pipeline parameters displayed approximately uniform distributions over the ranges defined in table Table B1 as expected.

Symptom enrichment by cluster is provided in Supplemental Data 3, reproduced as Table B2 below and summarized in Table B3.

References

Boongoen T, Iam-On N. Cluster ensembles: A survey of approaches with recent extensions and applications. *Comput Sci Rev* 2018;**28**:1–25.

Fern XZ, Lin W. Cluster Ensemble Selection. *Stat Anal Data Min ASA Data Sci J* 2008;**1**:128–41.

von Luxburg U. A tutorial on spectral clustering. *Stat Comput* 2007;**17**:395–416.

Zelnik-Manor L, Perona P. Self-tuning spectral clustering. *Proceedings of the 17th International Conference on Neural Information Processing Systems*. Cambridge, MA, USA: MIT Press, 2004, 1601–8.

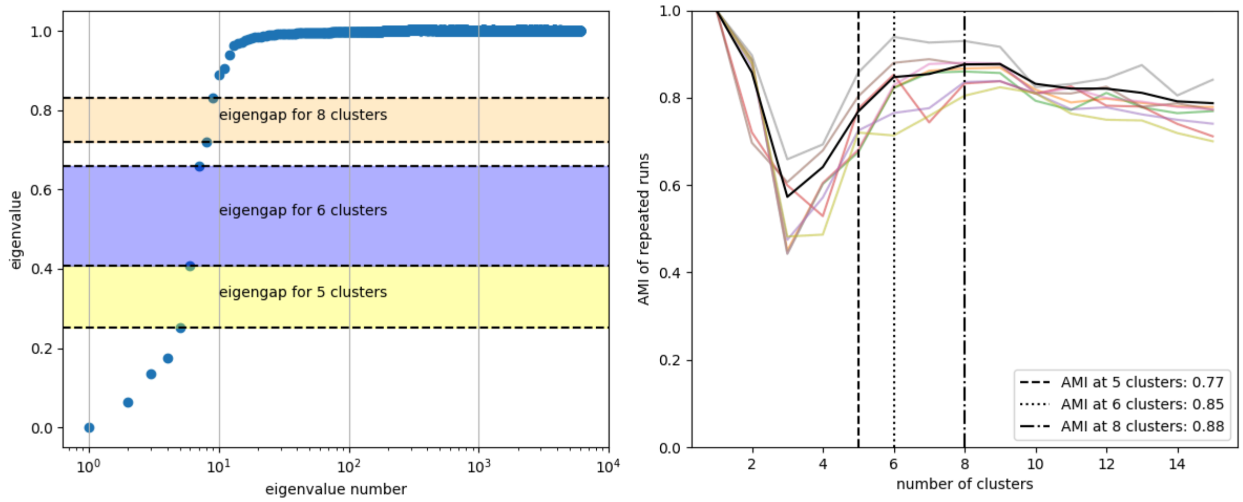

Figure B1: Left: illustration of the eigengaps of the normalized graph Laplacian produced by the co-association matrix of the selected ensemble, which were used to select candidates for the best number of clusters to use. Right: the stability of the cluster structure (for 1 to 15 clusters) under repeated runs, calculated as AMI value with first run. Each coloured line is a separate run of the full methodology, the solid black line is the mean over these runs. The three dashed lines indicate the location of the three highest eignegaps from the left panel at 5, 6, and 8 clusters.

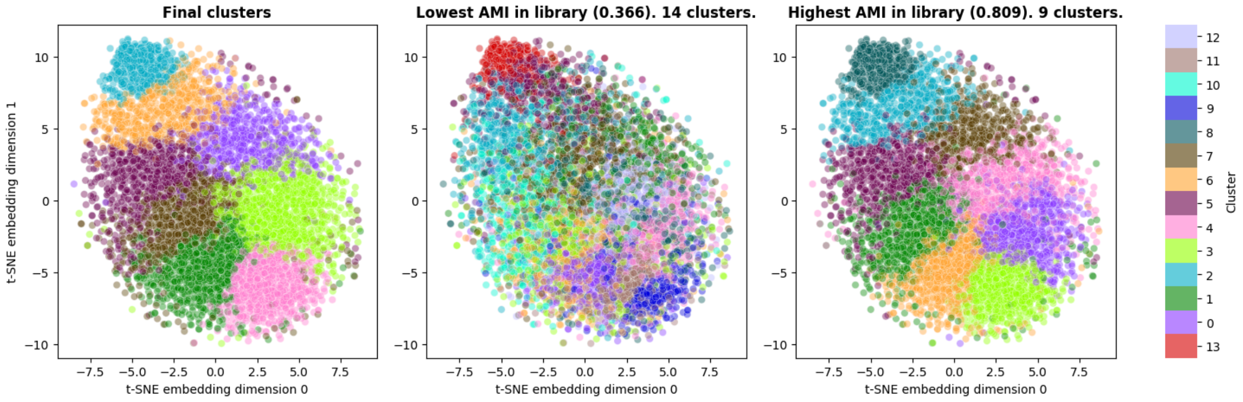

Figure B2: Embeddings of the symptom data with patients coloured by cluster, using the same t-SNE embedding as Fig 1 in the main text. Right: final clusters ('clustering B'). Middle: library clustering with lowest AMI with final clusters. Right: library clustering with highest AMI with final clusters.

Algorithm	Parameter	Search space

PCA	n_components	(2, 40)
HDBSCAN	cluster_selection_method	[‘eom’, ‘leaf’]
HDBSCAN	metric	['euclidean', 'manhattan']
HDBSCAN	min_cluster_size	(2, 100)
HDBSCAN	min_samples	(1, 50)
UMAP	n_neighbors	(2, 1000)
UMAP	min_dist	(0, 1)
UMAP	n_components	(2, 40)
UMAP	metric	['euclidean', 'manhattan']
K-MEANS	n_clusters	(2, 20)
K-MEANS	n_init	(1, 10)
K-MEANS	init	['k-means++', 'random']

Table B1: Parameter search space used for initial experiments with library generation using Bayes Search and Randomized search.

Table B2: The following table shows the full set of symptoms, their frequency in the whole population and each cluster, as well as the difference δ between the cluster and population frequencies. Enriched symptoms, highlighted in blue, are those for which $\delta > 0.1$. Conversely dis-enriched symptoms, in orange, have $\delta < -0.1$.

Symptom	Population	0		1		2		3		4		5		6		7	
		δ	f	δ	f	δ	f	δ	f	δ	f	δ	f	δ	f	δ	f
Fatigue	0.979	0.021	1	-0.012	0.968	0.017	0.996	0.018	0.997	-0.07	0.91	0.014	0.993	0.016	0.995	0.016	0.996
Memory_short	0.924	0.035	0.959	0.005	0.929	0.044	0.968	-0.021	0.903	-0.028	0.896	-0.001	0.923	0.038	0.962	-0.032	0.893
PEM	0.884	0.084	0.967	-0.005	0.878	0.11	0.994	0.03	0.914	-0.308	0.576	0.074	0.957	0.097	0.98	0.033	0.917
Cognitive_Functioning_attentionconcentration	0.753	0.216	0.969	-0.365	0.388	0.229	0.982	0.167	0.92	-0.356	0.397	0.125	0.878	0.191	0.944	-0.01	0.743
Shortness_of_breath	0.737	0.105	0.842	0.088	0.825	0.192	0.929	-0.114	0.623	-0.436	0.301	0.134	0.871	0.167	0.903	0.106	0.843
Insomnia	0.693	0.084	0.777	-0.12	0.573	0.269	0.962	-0.087	0.606	-0.298	0.396	0.193	0.886	0.21	0.903	0.012	0.705
Tightness_of_chest	0.68	0.061	0.741	0.128	0.808	0.237	0.917	-0.172	0.508	-0.443	0.237	0.167	0.847	0.194	0.874	0.105	0.785
Muscle_aches	0.677	0.094	0.771	-0.166	0.511	0.297	0.974	-0.107	0.57	-0.311	0.366	0.188	0.865	0.231	0.908	0.074	0.751
Dizziness_vertigo	0.673	0.147	0.82	-0.206	0.467	0.298	0.97	-0.086	0.586	-0.339	0.334	0.194	0.867	0.277	0.949	0.034	0.706
Cognitive_Functioning_thinking	0.666	0.295	0.961	-0.464	0.202	0.3	0.966	0.244	0.91	-0.427	0.239	0.111	0.777	0.242	0.908	-0.067	0.599
Heart_palpitations	0.656	0.127	0.784	0.004	0.661	0.255	0.911	-0.171	0.485	-0.38	0.276	0.206	0.862	0.231	0.887	0.036	0.692
Dry_cough	0.636	0.091	0.727	-0.012	0.624	0.188	0.824	-0.104	0.532	-0.267	0.369	0.055	0.691	0.133	0.769	0.127	0.763
Tachycardia	0.592	0.068	0.66	-0.009	0.584	0.315	0.907	-0.169	0.424	-0.367	0.225	0.184	0.776	0.262	0.854	0.035	0.627
Cognitive_Functioning_executive	0.589	0.346	0.935	-0.464	0.125	0.336	0.925	0.26	0.849	-0.42	0.169	0.051	0.64	0.256	0.845	-0.124	0.465
Gasping_air_normal_oxygen	0.566	0.091	0.657	0.04	0.607	0.258	0.824	-0.163	0.404	-0.416	0.15	0.175	0.741	0.216	0.782	0.096	0.662
Cognitive_Functioning_problemsolving	0.557	0.337	0.894	-0.43	0.127	0.36	0.917	0.241	0.798	-0.425	0.132	0.008	0.565	0.299	0.856	-0.132	0.425
Sore_throat	0.552	0.082	0.634	-0.033	0.518	0.326	0.877	-0.182	0.37	-0.274	0.277	0.124	0.675	0.176	0.728	0.085	0.637
Diarrhea	0.551	-0.005	0.546	-0.035	0.516	0.297	0.848	-0.214	0.338	-0.258	0.293	0.187	0.739	0.246	0.797	0.089	0.64
Elevated_temp	0.55	0.103	0.654	-0.172	0.378	0.276	0.826	-0.294	0.256	-0.266	0.284	0.203	0.754	0.253	0.804	0.29	0.84
Joint_pain	0.547	0.066	0.613	-0.162	0.385	0.392	0.939	-0.151	0.395	-0.26	0.286	0.182	0.729	0.291	0.838	-0.014	0.532
Chills_flushing_sweats	0.543	0.091	0.634	-0.159	0.384	0.374	0.917	-0.265	0.278	-0.313	0.23	0.193	0.736	0.308	0.851	0.189	0.732
Sleep_other	0.528	0.135	0.663	-0.157	0.372	0.286	0.814	-0.074	0.454	-0.244	0.284	0.126	0.655	0.197	0.725	-0.006	0.523
Loss_of_appetite	0.499	0.06	0.559	-0.095	0.404	0.331	0.83	-0.189	0.31	-0.249	0.25	0.143	0.642	0.196	0.696	0.118	0.617
Speech_difficulty_finding_words	0.485	0.492	0.977	-0.322	0.163	0.464	0.949	0.065	0.55	-0.301	0.184	-0.178	0.307	0.338	0.823	-0.195	0.29
Waking_up_in_night	0.483	0.118	0.602	-0.144	0.339	0.293	0.777	-0.102	0.381	-0.263	0.22	0.16	0.644	0.214	0.697	0.005	0.489
Sensations_tingling_prickling	0.481	-0.048	0.433	-0.091	0.39	0.404	0.885	-0.139	0.342	-0.235	0.246	0.194	0.675	0.323	0.804	-0.101	0.38
Pain_burning_chest	0.473	0.015	0.488	0.095	0.568	0.355	0.828	-0.219	0.254	-0.378	0.095	0.189	0.662	0.211	0.684	0.055	0.528
Nausea	0.464	0.069	0.533	-0.135	0.329	0.412	0.875	-0.201	0.262	-0.289	0.175	0.214	0.678	0.279	0.743	0.028	0.492
Sensations_weakness	0.462	0.074	0.537	-0.153	0.31	0.382	0.844	-0.084	0.378	-0.246	0.216	0.118	0.58	0.266	0.728	-0.061	0.401
Sensations_skin_burning_no_rash	0.457	-0.044	0.413	-0.156	0.302	0.466	0.923	-0.153	0.304	-0.189	0.268	0.18	0.637	0.353	0.81	-0.115	0.342
Cognitive_Functioning_slowedthoughts	0.43	0.295	0.725	-0.256	0.174	0.325	0.755	0.067	0.498	-0.254	0.176	-0.012	0.418	0.215	0.645	-0.132	0.298
Memory_long	0.415	0.349	0.764	-0.337	0.078	0.415	0.83	0.111	0.526	-0.261	0.154	-0.088	0.327	0.243	0.658	-0.15	0.266
Gastrointestinal_abdominal_pain	0.413	-0.008	0.405	-0.081	0.333	0.47	0.883	-0.205	0.208	-0.249	0.165	0.192	0.605	0.269	0.682	-0.028	0.386
Vibrating_sensations	0.41	-0.028	0.382	-0.072	0.338	0.406	0.816	-0.153	0.257	-0.232	0.178	0.208	0.618	0.287	0.697	-0.11	0.299
Difficulty_falling_asleep	0.406	0.128	0.533	-0.133	0.273	0.324	0.729	-0.101	0.305	-0.233	0.172	0.12	0.525	0.216	0.622	-0.04	0.366
Tremors	0.403	-0.018	0.385	-0.063	0.341	0.421	0.824	-0.154	0.25	-0.229	0.175	0.173	0.576	0.317	0.72	-0.132	0.271
Headaches_behind_eyes	0.402	0.021	0.423	-0.17	0.232	0.365	0.767	-0.052	0.349	-0.183	0.219	0.164	0.565	0.178	0.579	-0.08	0.322
Night_sweats	0.4	0.05	0.45	-0.162	0.239	0.384	0.785	-0.151	0.25	-0.25	0.15	0.199	0.6	0.241	0.642	0.018	0.418
Headaches_stiff_neck	0.4	0.067	0.467	-0.188	0.212	0.438	0.838	-0.097	0.303	-0.229	0.171	0.126	0.525	0.284	0.684	-0.076	0.323
Loss_of_smell	0.392	0.057	0.449	-0.196	0.196	0.163	0.555	-0.047	0.345	0.053	0.445	-0.087	0.305	0.063	0.455	0.118	0.51
Merged_temperature_other_temp_issues	0.391	0.064	0.455	-0.04	0.351	0.277	0.668	-0.127	0.264	-0.19	0.201	0.111	0.502	0.167	0.558	-0.027	0.364
Temp_lability	0.379	0.071	0.45	-0.173	0.206	0.287	0.666	-0.213	0.167	-0.203	0.176	0.098	0.477	0.191	0.57	0.266	0.645
Eye_Vision_blurred_vision	0.371	0.112	0.483	-0.181	0.19	0.489	0.86	-0.113	0.258	-0.246	0.125	0.095	0.466	0.316	0.687	-0.11	0.261
Sensations_numbness_loss_sensation	0.362	-0.053	0.309	-0.119	0.243	0.45	0.812	-0.109	0.253	-0.202	0.16	0.155	0.517	0.287	0.65	-0.112	0.25
Loss_of_taste	0.358	0.05	0.408	-0.174	0.185	0.203	0.561	-0.06	0.299	0.024	0.382	-0.076	0.282	0.058	0.416	0.116	0.475
Sensations_coldness	0.353	-0.019	0.333	-0.129	0.224	0.434	0.787	-0.132	0.221	-0.198	0.154	0.107	0.459	0.286	0.638	-0.038	0.315
Ear_sensitivity_to_noise	0.351	0.099	0.45	-0.199	0.153	0.493	0.844	-0.067	0.284	-0.211	0.14	0.044	0.395	0.294	0.645	-0.122	0.229
Headaches_temples	0.348	0.045	0.393	-0.151	0.197	0.262	0.611	-0.058	0.29	-0.142	0.206	0.103	0.451	0.159	0.507	-0.015	0.333
Eye_vision_symptoms	0.347	0.071	0.418	-0.162	0.185	0.497	0.844	-0.101	0.246	-0.222	0.125	0.078	0.425	0.314	0.661	-0.134	0.213
Headaches_diffuse	0.344	0.048	0.392	-0.131	0.212	0.303	0.646	-0.002	0.341	-0.183	0.16	0.087	0.431	0.111	0.455	-0.048	0.295
Acid_reflux	0.341	0.018	0.359	-0.029	0.312	0.35	0.692	-0.16	0.181	-0.196	0.145	0.136	0.477	0.158	0.499	-0.011	0.331
Tinnitus	0.341	-0.008	0.333	-0.104	0.237	0.449	0.791	-0.088	0.253	-0.18	0.161	0.07	0.411	0.243	0.584	-0.107	0.234
Headaches_brain_pressure	0.336	0.04	0.376	-0.198	0.138	0.443	0.779	-0.034	0.302	-0.21	0.126	0.116	0.453	0.242	0.578	-0.119	0.218
Muscle_spasms	0.334	0.009	0.343	-0.137	0.197	0.492	0.826	-0.146	0.188	-0.217	0.117	0.143	0.477	0.306	0.64	-0.102	0.232
Awakened_cannot_breathe	0.331	0.011	0.341	-0.065	0.266	0.365	0.696	-0.16	0.171	-0.262	0.069	0.197	0.528	0.227	0.558	-0.01	0.321
Runny_nose	0.328	0.045	0.372	-0.104	0.224	0.301	0.628	-0.102	0.225	-0.09	0.238	0.046	0.374	0.114	0.442	0.001	0.329
Extreme_thirst	0.327	-0.004	0.324	-0.082	0.245	0.412	0.739	-0.129	0.198	-0.217	0.11	0.113	0.44	0.226	0.553	-0.028	0.299
Vivid_dreams	0.321	0.074	0.395	-0.145	0.175	0.335	0.656	-0.048	0.272	-0.187	0.134	0.104	0.425	0.17	0.491	-0.069	0.251
SkinAllergy_itchy_skin	0.318	-0.027	0.291	-0.139	0.179	0.456	0.775	-0.117	0.201	-0.139	0.179	0.052	0.37	0.276	0.594	-0.064	0.254
Neuralgia	0.314	-0.044	0.27	-0.124	0.19	0.518	0.832	-0.142	0.173	-0.206	0.108	0.144	0.458	0.303	0.617	-0.108	0.206
Eye_Vision_sensitivity_to_light	0.314	0.102	0.416	-0.211	0.103	0.552	0.866	-0.091	0.223	-0.224	0.09	0.06	0.374	0.305	0.619	-0.126	0.188
Gastrointestinal_feeling_full_quickly_eating	0.312	0.021	0.333	-0.056	0.257	0.314	0.626	-0.133	0.179	-0.146	0.167	0.1	0.413	0.157	0.47	-0.023	0.29
Speech_difficulty_communicating_verbally	0.306	0.477	0.784	-0.253	0.053	0.502	0.808	0.02	0.327	-0.237	0.069	-0.199	0.107	0.245	0.552	-0.203	0.103
Difficulty_swallowing	0.306	-0.024	0.281	-0.068	0.237	0.437	0.743	-0.11	0.195	-0.209	0.097	0.075	0.381	0.228	0.534	-0.049	0.257
Fever	0.299	0.09	0.389	-0.142	0.157	0.209	0.508	-0.182	0.117	-0.182	0.117	0.07	0.369	0.161	0.46	0.249	0.548
Cough_with_mucus	0.291	0.036	0.327	-0.042	0.249	0.209	0.5	-0.096	0.195	-0.104	0.187	0.017	0.308	0.107	0.398	0.041	0.332
Eye_Vision_dry_eyes	0.288	-0.021	0.267	-0.088	0.2	0.414	0.702	-0.088	0.2	-0.162	0.126	0.046	0.334	0.19	0.478	-0.041	0.247
Headaches_base_of_skull	0.282	0.021	0.302	-0.133	0.149	0.355	0.636	-0.074	0.208	-0.163	0.118	0.11	0.392	0.208	0.489	-0.081	0.201
Altered_taste	0.279	0.09	0.369	-0.131	0.148	0.338	0.617	-0.086	0.193	-0.013	0.266	-0.042	0.237	0.081	0.36	-0.033	0.246
Heat_intolerance	0.277	0.043	0.32	-0.142	0.135	0.409	0.686	-0.122	0.156	-0.176	0.101	0.085	0.362	0.255	0.532	-0.051	0.226
Sneezing	0.273	0	0.273	-0.086	0.187	0.387	0.66	-0.104	0.169	-0.119	0.154	0.028	0.301	0.126	0.399	0.004	0.277
Headaches_after_mental_exertion	0.268	0.151	0.42	-0.197	0.071	0.412	0.68	-0.035	0.233	-0.223	0.045	0.063	0.331	0.215	0.483	-0.1	0.168
Skin_rashes	0.263	-0.019	0.244	-0.057	0.206	0.367	0.63	-0.089	0.174	-0.131	0.132	0.028	0.292	0.161	0.424	-0.04	0.223
Speech_difficulty_understanding_others_speech	0.26	0.431	0.691	-0.246	0.014	0.531	0.791	-0.014	0.246	-0.221	0.038	-0.191	0.069	0.262	0.522	-0.183	0.076
Speech_difficulty_reading_processing_text	0.259	0.417	0.676	-0.232	0.028	0.513	0.773	-0.01	0.25	-0.222	0.037	-0.167	0.092	0.269	0.529	-0.21	0.049
Eye_Vision_eye_pressure_pain	0.259	0.011	0.27	-0.137	0.123	0.476	0.735	-0.114	0.146	-0.165	0.095	0.082	0.341	0.212	0.471	-0.064	0.195
Constipation	0.258	0.018	0.276	-0.07	0.188	0.291	0.549	-0.096	0.162	-0.142	0.116	0.068	0.326	0.21	0.468	-0.06	0.198
Eye_Vision_other_eye_issues	0.251	0.036	0.288	-0.051	0.201	0.264	0.516	-0.054	0.197	-0.089	0.162	0.006	0.257	0.096	0.347	-0.051	0.201
Bone_ache_burning	0.251	-0.018	0.233	-0.12	0.131	0.421	0.672	-0.124	0.127	-0.161	0.09	0.104	0.355	0.243	0.494	-0.059	0.192
Sensations_electrical_zaps	0.251	-0.044	0.207	-0.101	0.15	0.447	0.698	-0.111	0.14	-0.142	0.109	0.103	0.354	0.238	0.489	-0.118	0.133
Changes_in_voice	0.251	-0.023	0.228	-0.043	0.208	0.301	0.551	-0.071	0.179	-0.149	0.101	0.018	0.268	0.17	0.421	-0.009	0.242
Phantom_smell	0.249	0.042	0.291	-0.136	0.112	0.395	0.644	-0.108	0.141	-0.039	0.21	-0.026	0.223	0.128	0.376	-0.017	0.232
Ear_ear_pain	0.247	-0.039	0.208	-0.078	0.169	0.449	0.696	-0.108	0.138	-0.169	0.078	0.083	0.33	0.162	0.409	-0.035	0.212
Waking_up_early	0.246	0.059	0.306	-0.079	0.167	0.226	0.472	-0.063	0.183	-0.129	0.117	0.058	0.304	0.115	0.362	-0.015	0.232
SkinAllergy_itchy_eyes	0.245	-0.026	0.22	-0.121	0.124	0.49	0.735	-0.106	0.139	-0.119	0.126	0.036	0.281	0.185	0.43	-0.061	0.184
Headaches_migraines	0.241	0.009	0.25	-0.123	0.118	0.306	0.547	-0.037	0.205	-0.144	0.097	0.094	0.336	0.148	0.39	-0.061	0.181
Nightmares	0.241	0.05	0.291	-0.105	0.136	0.352	0.593	-0.076	0.166	-0.163	0.078	0.059	0.3	0.168	0.409	-0.048	0.194
Gastrointestinal_hyperactive_bowel	0.239	-0.04	0.198	-0.086	0.153	0.305	0.543	-0.113	0.126	-0.125	0.114	0.09	0.329	0.198	0.437	-0.008	0.23
Speech_difficulty_speaking_complete_sentences	0.234	0.33	0.564	-0.202	0.032	0.479	0.713	0.011	0.245	-0.197	0.037	-0.158	0.076	0.201	0.435	-0.162	0.072
Altered_smell	0.227	0.053	0.28	-0.134	0.093	0.297	0.524	-0.081	0.146	0.028	0.255	-0.03	0.197	0.053	0.28	-0.016	0.21
Ear_changes_to_ear_canal	0.216	-0.056	0.159	-0.07	0.146	0.395	0.611	-0.096	0.119	-0.106	0.109	0.064	0.279	0.128	0.344	-0.04	0.175
Change_sensitivity_to_meds	0.209	0.001	0.21	-0.046	0.163	0.263	0.472	-0.053	0.156	-0.104	0.105	0.05	0.259	0.112	0.321	-0.066	0.143
Other_ear_hearing_symptoms	0.207	0.006	0.213	-0.053	0.155	0.283	0.49	-0.051	0.157	-0.078	0.13	0.009	0.216	0.069	0.277	-0.034	0.174
Eye_Vision_floaters	0.204	0.003	0.207	-0.092	0.112	0.381	0.585	-0.079	0.125	-0.131	0.073	0.045	0.249	0.172	0.376	-0.07	0.134
Low_temp	0.194	-0.012	0.182	-0.06	0.134	0.326	0.52	-0.12	0.074	-0.124	0.07	0.07	0.264	0.163	0.357	-0.018	0.177
Acute_confusion	0.194	0.049	0.242	-0.155	0.039	0.451	0.644	-0.038	0.156	-0.157	0.036	-0.006	0.187	0.176	0.37	-0.064	0.13
Restless_leg	0.19	0.013	0.203	-0.091	0.099	0.273	0.462	-0.062	0.128	-0.118	0.072	0.049	0.239	0.183	0.373	-0.058	0.131
Reproductive_all_menstrual_issues	0.184	-0.011	0.172	-0.045	0.139	0.226	0.409	-0.073	0.11	-0.092	0.091	0.043	0.227	0.139	0.322	-0.031	0.153
Rattling_breath	0.175	0.015	0.19	-0.059	0.116	0.256	0.431	-0.083	0.092	-0.113	0.062	0.038	0.213	0.103	0.278	0.019	0.194
Cognitive_Functioning_fastthoughts	0.173	0.084	0.257	-0.115	0.058	0.22	0.393	-0.006	0.167	-0.102	0.071	0.007	0.18	0.094	0.267	-0.039	0.134
Speech_difficulty_communicating_writing	0.167	0.273	0.441	-0.147	0.021	0.435	0.603	-0.015	0.152	-0.149	0.018	-0.127	0.04	0.137	0.304	-0.145	0.023

	Most Enriched	Most dis-enriched	Cluster Name
0	Speech_difficulty_finding_words<br>Speech_difficulty_communicating_verbally,<br>Speech_difficulty_understanding_others_speech<br>Speech_difficulty_reading_processing_text<br>Memory_long Cognitive_Functioning_executive<br>Cognitive_Functioning_problemsolving<br>Speech_difficulty_speaking_complete_sentencs<br>Cognitive_Functioning_slowedthoughts<br>Cognitive_Functioning_thinking	Inability_to_yawn<br>Ear_numbness<br>Coughing_up_blood<br>New_anaphylaxis<br>Reproductive_testicle_penis_pain_changes<br>Hallucinations_tactile<br>Sensations_facial_paralysis<br>SkinAllergy_shingles<br>Hallucinations_other<br>Reproductive_postmenopausal_bleeding	Speech, memory, cognition, sleep and eye/vision (neurological).
1	Tightness_of_chest<br>Pain_burning_chest<br>Shortness_of_breath<br>Gasping_air_normal_oxygen<br>Memory_short<br>Heart_palpitations<br>Respiratory_other	Eye_Vision_tunnel_vision<br>Reproductive_testicle_penis_pain_changes<br>Hallucinations_tactile<br>Sensations_facial_paralysis<br>Heightened_taste<br>SkinAllergy_shingles<br>Hallucinations_other<br>Reproductive_postmenopausal_bleeding<br>Reproductive_early_menopause<br>Eye_Vision_loss_of_vision	Shortness of breath, chest burning pain, short term memory, heart palpitations.
2	Eye_Vision_sensitivity_to_light<br>Speech_difficulty_understanding_others_speech<br>Neuralgia<br>Speech_difficulty_reading_processing_text<br>Speech_difficulty_communicating_verbally<br>Eye_vision_symptoms<br>Ear_sensitivity_to_noise<br>Muscle_spasms<br>SkinAllergy_itchy_eyes<br>Eye_Vision_blurred_vision		Sensory sensitivity, difficulty communicating verbally and processing information, neuropathy, itchy eyes and blurred vision.
3	Cognitive_Functioning_executive<br>Cognitive_Functioning_thinking<br>Cognitive_Functioning_problemsolving<br>Cognitive_Functioning_attentionconcentration<br>Memory_long<br>Cognitive_Functioning_slowedthoughts<br>Speech_difficulty_finding_words<br>PEM<br>Speech_difficulty_communicating_verbally<br>Fatigue	Eye_Vision_tunnel_vision<br>Reproductive_testicle_penis_pain_changes<br>Hallucinations_tactile<br>Sensations_facial_paralysis<br>Heightened_taste<br>SkinAllergy_shingles<br>Hallucinations_other<br>Reproductive_postmenopausal_bleeding<br>Reproductive_early_menopause<br>Eye_Vision_loss_of_vision	Cognition, long term memory, speech, PEM, and fatigue.
4	Loss_of_smell<br>Altered_smell<br>Loss_of_taste<br>Respiratory_other	Eye_Vision_tunnel_vision<br>Reproductive_testicle_penis_pain_changes<br>Hallucinations_tactile<br>Sensations_facial_paralysis<br>Heightened_taste<br>SkinAllergy_shingles<br>Hallucinations_other<br>Reproductive_postmenopausal_bleeding<br>Reproductive_early_menopause<br>Eye_Vision_loss_of_vision	Altered smell and taste, respiratory.
5	Nausea<br>Vibrating_sensations Heart_palpitations<br>Elevated_temp 'Night_sweats<br>Awakened_cannot_breathe	Cognitive_Functioning_other<br>Respiratory_other<br>Heightened_smell<br>Hallucinations_auditory	Nausea, temperature dysregulation, sleep disturbance, paresthesia and

	Sensations_tingling_prickling Dizziness_vertigo<br>Chills_flushing_sweats<br>Insomnia	SkinAllergy_other_itchy<br>Speech_changes_to_secondary_languages<br>Speech_other<br>Eye_Vision_tunnel_vision<br>Heightened_taste<br>Reproductive_postmenopausal_bleeding	vibrations, palpitations, dizziness and vertigo.
6	Sensations_skin_burning_no_rash<br>Speech_difficulty_finding_words<br>Sensations_tingling_prickling<br>Tremors<br>Eye_Vision_blurred_vision<br>Eye_vision_symptoms<br>Chills_flushing_sweats<br>Muscle_spasms<br>Eye_Vision_sensitivity_to_light<br>Neuralgia	Respiratory_other<br>SkinAllergy_shingles	Paresthesia, speech, vision problems, neuropathy, tremors, temperature dysregulation.
7	Elevated_temp<br>Temp_lability<br>Fever<br>Chills_flushing_sweats<br>Dry_cough<br>Loss_of_appetite<br>Loss_of_smell<br>Loss_of_taste<br>Shortness_of_breath<br>Tightness_of_chest	Speech_other<br>New_anaphylaxis<br>Eye_Vision_tunnel_vision<br>Reproductive_testicle_penis_pain_changes<br>Hallucinations_tactile<br>Sensations_facial_paralysis<br>Heightened_taste<br>SkinAllergy_shingles<br>Reproductive_early_menopause<br>Eye_Vision_loss_of_vision	Temperature dysregulation, dry cough, loss of smell, taste and appetite, shortness of breath.

Table B3: The most enriched and dis-enriched symptoms for each cluster up to a maximum of 10 symptoms. Names were allocated to each cluster by the research team based on these symptoms.

Supplementary Note C: Supplemental information for method C.

Latent class analysis is a probabilistic model-based clustering method that identifies groups of related cases within a heterogeneous population (Sinha, Calfee and Delucchi 2021). Patients are assumed to be sampled from a finite mixture of *k* latent classes, with each class characterized by the conditional probabilities that patients in that class experience each recorded symptom. The model learns likely classes and the parameters of those classes (symptom probabilities), which can then be used to probabilistically predict the class membership of patients. By modeling class membership probabilities, patients could be assigned to the most likely latent class.

Latent class analysis (LCA) was performed using StepMix (Morin *et al.* 2024) v. 2.1.3 in Python 3.12. Default parameter settings were used when not otherwise specified. Symptoms present in more than 95% or fewer than 5% of patients were removed. Grid search was performed over 2–25 clusters, with the Bayesian information criterion (BIC) computed for each cluster count. The optimal BIC was 13 clusters for the single run and an average of 13 clusters when comparing across ten random seeds, so n_components was set to 13.

For the consensus clustering, which defines clusters using commonalities between ten clusterings with different random seeds, consensus was determined using a co-association matrix as described for method B above. Hierarchical clustering was performed on the vector of LCA clusters for each patient using scipy v. 1.11.3 `fcluster` with criterion distance and threshold 0.026, with the threshold chosen such that the number of clusters was maintained at 13. tSNE plots of the LCA clustering were created using scikit-learn v. 1.3.2 function TSNE with default parameters. The input data used for tSNE were the 13 cluster membership probabilities for each patient as outputted from single-run LCA.

For robustness analysis, the model was trained using 80% sub-samplings of patients ten times. Clusters were then assigned to all points using the StepMix function `predict`. The AMI of the full dataset was compared across runs. For further evaluation and exploration of performance in settings with fewer symptom counts, symptoms were subsampled to 0.1–0.9 of reported symptoms and a grid search was performed for 1–20 clusters for random seeds 1–10.

In contrast to methods A and B, to identify symptoms characterizing each cluster, we examined the parameters of the mixture model corresponding to each symptom as produced by the StepMix function `get_mm_df`. The value for each parameter corresponds to the probability that a patient in that cluster reported the relevant symptom. We define enriched and dis-enriched symptoms in the LCA model by subtracting the parameter value in the cluster of interest from the average parameter value for that symptom across all other clusters. Clusters had highly variable numbers of (dis-)enriched symptoms, so an per-cluster threshold was defined manually for describing cluster characteristics.

Results

The exact clusters produced by this method varied moderately with random seed selection, with an average AMI of 0.62 (Fig C1a). As such, we also produced a combined clustering where ten different maximum-likelihood estimate assignments were combined as for Clustering B, with the resulting co-association matrix hierarchically clustered to produce 13 groupings for comparison. The consensus clusters are visualized on the same tSNE plot produced using the single-run probabilistic assignment vectors (Fig C2c). To evaluate robustness to held-out patients, we repeated this procedure on ten random subsets of 80% of patients. The average pairwise AMI between these runs was 0.61, comparable to the difference between runs on the full dataset with different random seeds (Fig C1b). Model-derived symptom enrichment by cluster and associated symptom labelings is provided in Table C1.

We observe considerable overlap with the initial clustering (AMI 0.70), with the membership of 7 clusters of the original run maintaining at least 75% identity in the second (Fig C2c). For example, 98% of the patients assigned to C1 are assigned to consensus cluster 11. In contrast, 75% of patients in C0 are in the closest corresponding consensus cluster 4; however this subgroup of patients makes up 98% of consensus cluster 4, and are distinguished from the remainder of C0 by substantially decreased likelihood of reporting headaches after mental exertion (22% of C0 patients in consensus cluster 4 vs 46% of C0 patients not in consensus cluster 4), with pressure sensation (23% vs 45%), stiff neck (38% vs 61%), diffuse sensation (32% vs 54%), or migraine (22% vs 38%); as well as decreased reports of blurred vision (18% vs 40%) and light sensitivity (19% vs 39%).

The categorical similarity of these symptoms suggests that the consensus clustering has identified a true subgroup associated with migraine and headache symptoms, perhaps indicating a shared causal origin for these varied types of headaches that is connected with vision disturbances, and sometimes occurs alongside temperature dysregulation, sleep disturbance, and cognitive impairments. Notably, patients from some clusters which appear semantically similar (C2 and C11 are both characterized by speech and cognitive impairment) remain largely separate in the aggregated clustering, likely reflecting increased prevalence of sensory and vision-related symptoms in C2. Similarly, C8 and C10 are both characterized by high rates of insomnia, but also remain separate, with patients in C10 reporting more sensory symptoms, tremors, neuralgia and nausea. This suggests a possible pathological difference between generalized insomnia and insomnia accompanied by sensory abnormalities, in which such difference may be indicative of PEM phenotypes (Stussman *et al.* 2020).

References

Morin S, Legault R, Laliberté F *et al.* StepMix: A Python Package for Pseudo-Likelihood Estimation of Generalized Mixture Models with External Variables. 2024, DOI: 10.48550/arXiv.2304.03853.

Sinha P, Calfee CS, Delucchi KL. Practitioner's Guide to Latent Class Analysis: Methodological Considerations and Common Pitfalls. *Crit Care Med* 2021;**49**:e63–79.

Stussman B, Williams A, Snow J *et al.* Characterization of Post–exertional Malaise in Patients With Myalgic Encephalomyelitis/Chronic Fatigue Syndrome. *Front Neurol* 2020;**11**, DOI: 10.3389/fneur.2020.01025.

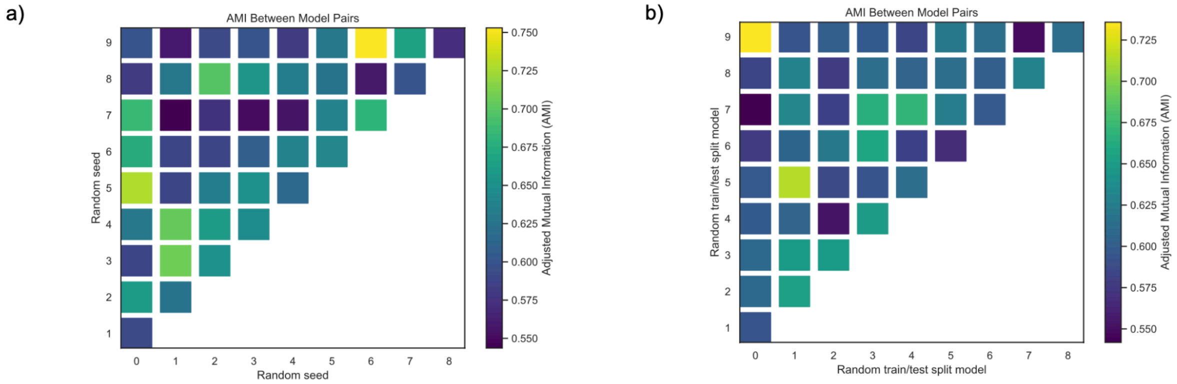

Figure C1: Supplemental information on model robustness a) AMI between models trained with different random seeds on the full dataset. b) AMI between models trained with different random subsets of the data, with the AMI computed on the full dataset.

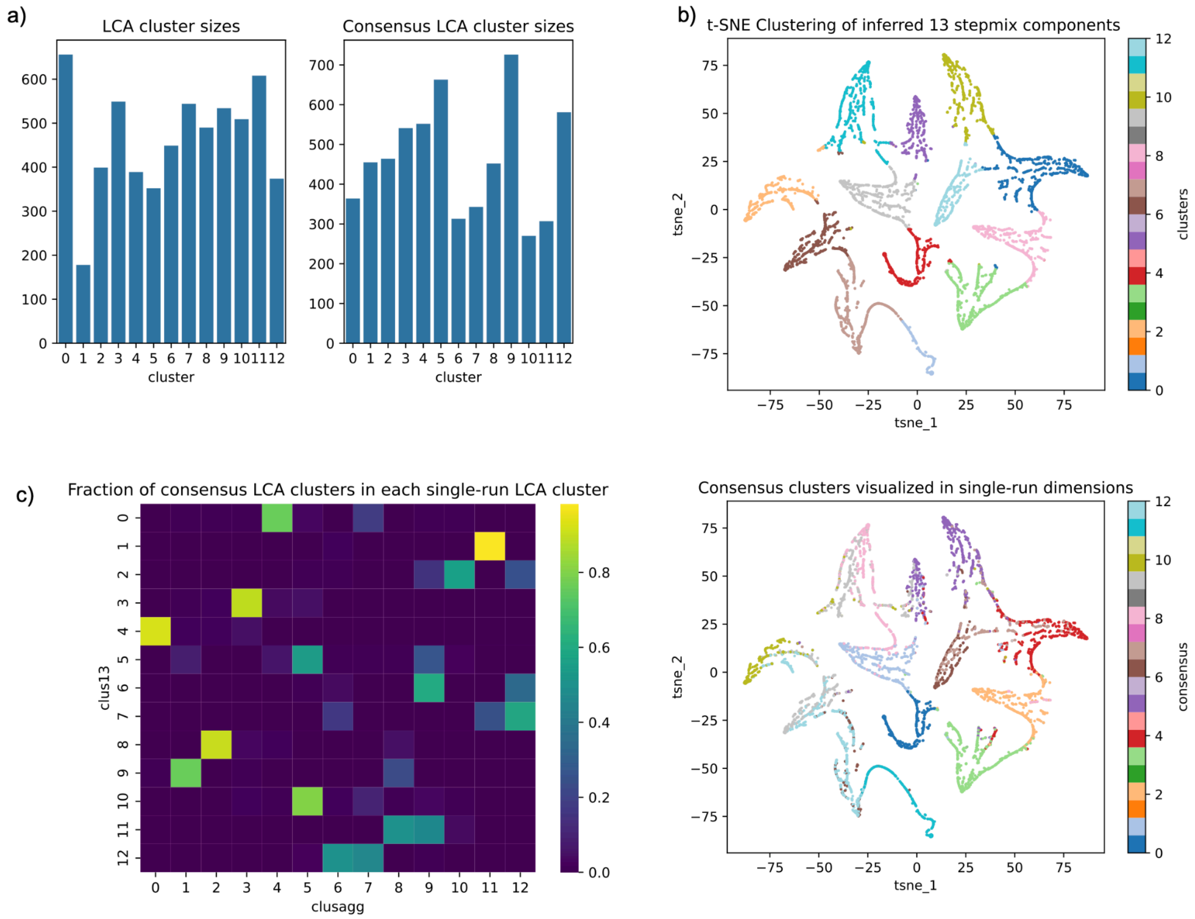

Figure C2: a) Cluster sizes for an individual LCA run presented as clustering C (left) and a consensus of ten LCA clusterings with different random seeds (right). Cluster numbers are arbitrary and do not indicate overlapping patient assignments. b) tSNE of probabilistic cluster assignments for all patients. Patients are colored by their most probable cluster. This visualization shows that clusters are well-defined, with only a few ambiguous assignments. c) Consensus clustering overlap (left) and on the same tSNE plot (right) visually clarify the substantial consensus across LCA clusterings.

Table C1: Cluster labeling information for Clustering C. Adaptive thresholds shown here are used for discussion purposes only, and were defined manually such that relevant symptoms fit on the page. Enriched symptoms marked with an asterisk (*) are dis-enriched and reported with reduced prevalence. Full parameter values are available in Supp Data 4.

C.	Name	Enriched symptoms	threshold	prevalent symptoms	threshold
0	Temperature dysregulation, sleep disturbance, cognition (attention/concentration) and shortness of breath	Insomnia, Elevated_temp, Waking_up_in_night, Fever, Temp_lability, Night_sweats, Cognitive_functioning_attentionconcentration	0.1	PEM, Memory_short, Insomnia, Cognitive-Functioning_attentionconcentration, Shortness_of_breath	0.8
1	High symptom burden with cognitive dysfunction, difficulty reading and communicating, and sensory and allergic symptoms Elevated musculoskeletal symptom count	Eye_Vision_sensitivity_to_light, Difficulty_swallowing, SkinAllergy_itchy_eyes, SkinAllergy_itchy_skin, Muscle_spasms, Speech_difficulty_reading_processing_text, Ear_pain, Speech_difficulty_understanding_others_speech, Sensations_numbness_loss_sensation	0.58	Cognitive_Functioning_thinking, Cognitibe_Functioning_attentionconcentration, PEM, Memory_short, Sensations_skin_burning_no_rash	0.98
2	Difficulty reading and communicating, high prevalence of cognitive symptoms	Speech_difficulty_finding_words, Speech_difficulty_communicating_verbally, Speech_difficulty_understanding_others_speech, Speech_dificulty_reading_processing_text, Speech_difficulty_speaking_complete_sentences	0.4	Speech_difficulty_finding_words, PEM, Cognitive_Functioning_attentionconcentration, Cognitive_function_thinking, Cognitive_Functioning_executive, Memory_short	0.95
3	Low symptom burden, cognitive symptoms reduced with short term memory difficulty still reported	Cognitive_Functioning_thinking*, Cognitive_Functioning_executive*, Cognitive_Functioning_attentionconcentration*, Cognitive_Functioning_problemsolving*,	-0.6	Memory_short, PEM	0.79

4	Lowest symptom burden, Lowest cognitive PEM severity, reduced frequency of PEM, cognitive symptoms reduced	Cognitive_Functioning_thinking*, Insomnia*, Cognitive_Functioning_attentionconcentration*, Cognitive_Functioning_executive*	-0.6	Memory_short (0.79), PEM (0.52)	0.5
5	Chest tightness, dizziness/vertigo, shortness of breath, gasping for air while oxygen normal	Gasping_air_normal_oxygen, tightness_of_chest, dizziness_vertigo, heart_palpitations	0.1	PEM, Memory_short, Shortness_of_breath, Tightness_of_chest, Dizziness_vertigo	0.8
6	Impaired reading and communication, elevated temperature and chills/sweats, sleep difficulty and elevated average musculoskeletal symptom count	Speech_difficulty_finding_words, Speech_difficulty_communicating_verbally, Speech_difficulty_reading_processing_text, Speech_difficulty_understanding-others_speech, Elevated_temp, Cognitive_Functionin_problemsolving, Difficulty_falling_asleep, Chills_flushing_sweats, Sleep_other	0.25	Speech_difficulty_finding_words, PEM, Cognitive_Functioning_attentionconcentration, Shortness_of_breath, Memory_short	0.9
7	High symptom burden with impaired speech, auditory and visual comprehension, blurred vision, pain: muscle aches, muscle spasms, neuralgia and skin burning without associated rash.	Speech_difficulty_finding_words, Speech_difficulty_communicating_verbally, Speech_difficulty_understanding-others_speech, Speech_difficulty_reading_processing_text,Eye_vision_symptoms, Speech_difficulty_speaking_complete_sentences, Muscle_spasms, Neuralgia, Sensations_skin_burning_no_rash, Blurred_vision	0.4	Symptom_Speech_difficulty_finding_words, PEM, Dizziness_vertigo, Memory_short, Muscle_aches	0.95
8	Insomnia, difficulty falling asleep and waking up in night	Insomnia, Waking_up_in_night, Difficulty_falling_asleep	0.1	Insomnia	1
9	Consistent sleepers with PEM and cognitive impairment	Difficulty_falling_asleep*, Waking_up_in_night*, Insomnia*	-0.4	Memory_short, PEM, Cognitive_Functioning_attentionconcentration, Cognitive_Functioning_thinking	0.7

10	Insomnia, tremors, sensory features: tingling/prickling, skin burning without rash, vibrations.	Insomnia, Sensations_tingling_prickling, Waking_up_in_night, Sensations_skin_burning_no_rash, Vibrating_sensations, Tremors	0.2	Insomnia	0.99
11	Speech and cognition with fewer symptoms per person	Speech_difficulty_finding_words, Speech_difficulty_communicating_verbally, Cognitive_functioning_executive, Speech_difficulty_speaking_complete_sentences	0.25	Speech_difficulty_finding_words, PEM, Memory_short, Cognitive_functioning_attentionconcentration, Cognitive_functioning_thinking	0.9
12	High burden with reduced cognitive/speech	Sensations_skin_burning_no_rash, Joint_pain, Neuralgia, Gastrointestinal_abdominal_pain, Sensations_tingling_prickling	0.28	PEM, Memory_short, Insomnia, Dizziness_vertigo, Muscle_aches	0.9

Supplementary Note D: Supplemental information for cluster comparison.

To produce the t-SNE embedding used to visualize the clusters in Fig 1 of the main text, we produced a set of N=500 randomly parameterised t-SNE embeddings of the symptom data by selecting parameters from the ranges given in table Supp Table D1. For each embedding we computed the silhouette score for clusterings A, B and C. The maximum silhouette scores across the 500 embeddings were -0.03, 0.24, 0.01 for clusterings A, B and C respectively. This suggested that it is easier to find (via random search) a 2D embedding where clustering B is well separated, than it is for clusterings A and C. For each of the 500 embeddings we calculated the mean silhouette score across the three clusterings, and selected the embedding that maximized this value. This produced an embedding with individual silhouette scores of -0.11, 0.24 and -0.05, which was found to have good clinical interpretability (Supplementary Fig 1 in the main text) and is the one chosen to represent the clusters in Fig 1 of the main text. To investigate alternative embeddings we defined a weighted average silhouette score as:

$$S_\alpha = (1 - \alpha)\, s_A + s_B + (1 - \alpha)\, s_C ,$$

where s_i is the raw silhouette score of embedding i and α is a weighting coefficient. We selected the embedding that maximized S_α for $\alpha \in [0, 0.5]$ and visualized the results in Supp Fig D1. The figure locates two candidate embeddings at α=0.50 (used in main text Fig 1 and Supp Fig 1), and α=0.27 (Supp Fig D2). It was notable that all of the embeddings examined showed approximately homogeneous densities in 2D with no clear structure visible, which supports our interpretation of the dataset in the main text. The same was true for the projection onto the first two principal components obtained using PCA, which is illustrated in Supp Fig D2.

The feature engineering of method A was able to produce a structured 2D embedding with a high degree of separation between clusters (Fig 2a of the main text), but with cluster assignments that were less similar in terms of AMI to clusterings B (0.13) and C (0.18). Similarly, probabilistic outputs of Method C could be used to produce a highly separated visualization (Fig 3b), but when clusterings A and B are visualized on the same axes, clusters are not well separated (Fig D4).

By comparing and combining three separate methods, we aimed to improve cluster robustness. However, each of the clustering methods used here come with their own set of drawbacks. The effectiveness of methodology A hinges greatly on data quality, as the dropping of features based on correlation may overlook important aspects in noisy datasets. Features that might appear redundant or less correlated in a noisy dataset might still hold significant predictive power once the noise is removed (Wagner et al., 1993). Through testing we found that the embeddings generated by the autoencoder were quite dependent on the initialized weights, potentially explaining this methodology producing the lowest AMI across runs. Future work applying this methodology could consider more refined feature selection techniques as well as methods to increase the stability of embeddings across autoencoder initialisation states. Method B uses k-means to generate the base clusterings for the ensemble. Although the k-means algorithm in

isolation is a weak clustering method, ensembles of k-means clusterings have been shown to be able to detect non-linearly separable and high-dimensional clusters (Wu *et al.* 2022). Nevertheless, it may be possible to improve on clustering B by using multiple algorithms to generate the base clusterings and experimenting with different consensus functions (Golalipour *et al.* 2021), and perhaps incorporating feature engineering similar to method A into the pipeline. Method C will perform best in cases where a mixture model is a good representation of the data (McLachlan and Lee 2019); if, for example, patients are better modeled as arising from overlapping phenotypes, where each patient could have more than one label, the single-cluster assumption may not hold. The mixture model used assumes a finite number of classes, that all observed patients fall into one of those classes, and that the classes themselves are homogenous (Sinha, Calfee and Delucchi 2021). If any of these assumptions do not hold, Method C may provide an inaccurate clustering.

References

Golalipour K, Akbari E, Hamidi SS *et al.* From clustering to clustering ensemble selection: A review. *Eng Appl Artif Intell* 2021;**104**:104388.

McLachlan GJ, Lee SX. Finite Mixture Models. *Annu Rev Stat Its Appl* 2019;**6**:355–78.

Sinha P, Calfee CS, Delucchi KL. Practitioner's Guide to Latent Class Analysis: Methodological Considerations and Common Pitfalls. *Crit Care Med* 2021;**49**:e63–79.

Wu H-F, Yu W, Saito-Diaz K *et al.* Norepinephrine transporter defects lead to sympathetic hyperactivity in Familial Dysautonomia models. *Nat Commun* 2022;**13**:7032.

**Parameter**	**Search space**
perplexity	[5, 100]
learning_rate	[10, 1000]
init	['random', 'pca']
metric	['euclidean', 'manhattan', 'hamming', 'jaccard', 'dice', 'russellrao', 'kulsinski', 'rogerstanimoto', 'sokalmichener', 'sokalsneath', 'yule']

Supplemental Table D1: Parameter ranges used to produce the set of 500 random t-SNE embeddings. For categorical parameters the search space is given as a list, for numerical parameters it is given as a closed interval on either $\mathbb{R}$ or $\mathbb{Z}$ as determined by the parameter.

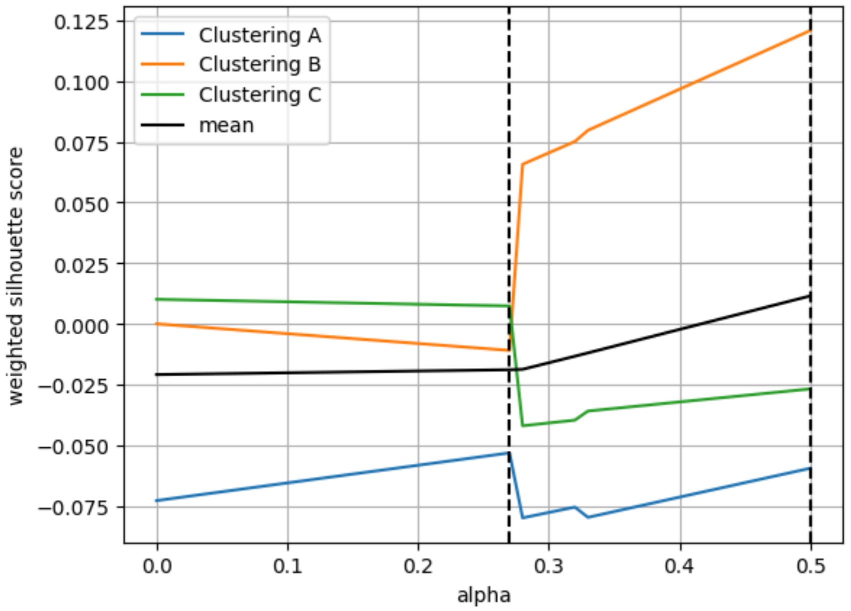

Supplemental Figure D1: The methodology for choosing a t-SNE embedding to plot all three clusterings by maximizing the weighted silhouette score (procedure described in the text of section SGM). The dashed line at α=0.5 indicates the embedding used in Fig 1 and Supp Fig 1 of the main text. The dashed line at α=0.27 indicates the alternative embedding illustrated in Supp Fig D2.

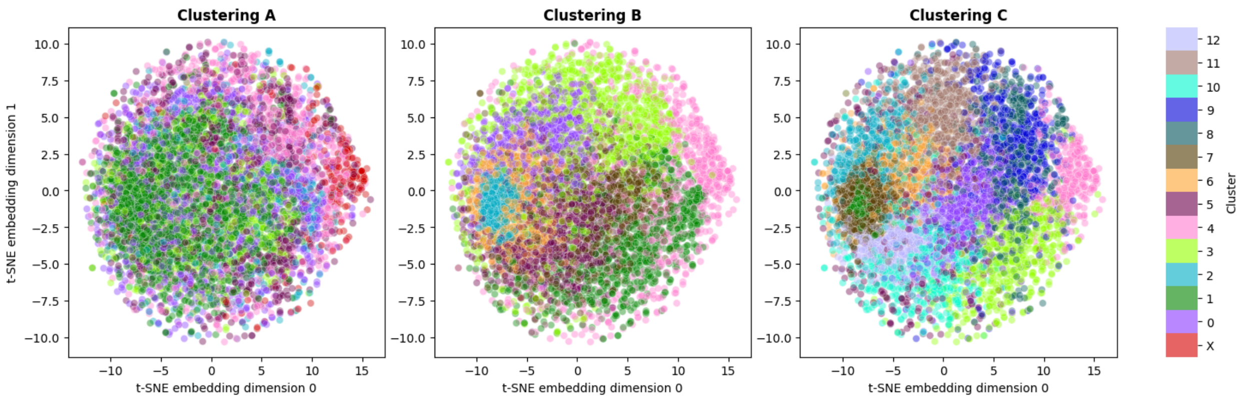

Supplemental Figure D2: Alternative t-SNE embedding of symptom data, selected at α=0.27 (see Supp Fig D1).

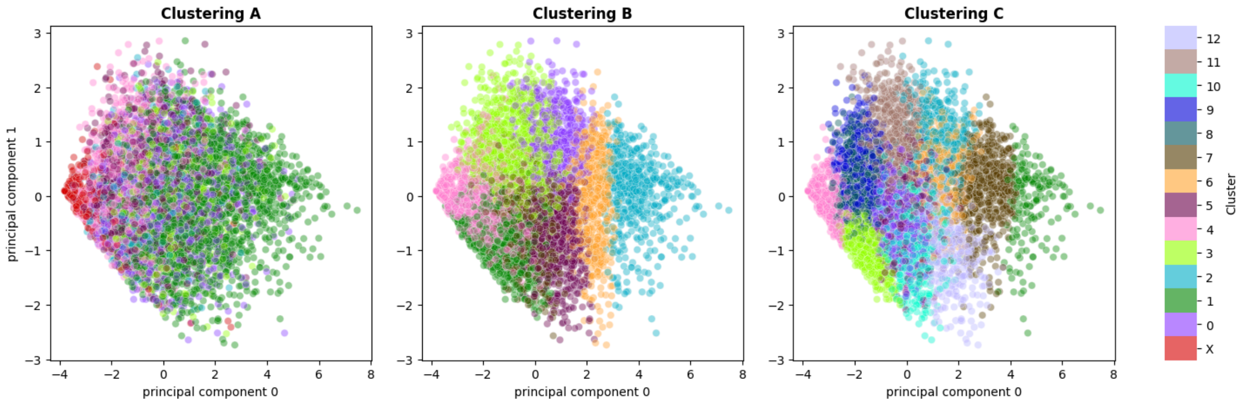

Figure D3: Projection of symptom data onto first two principal components.

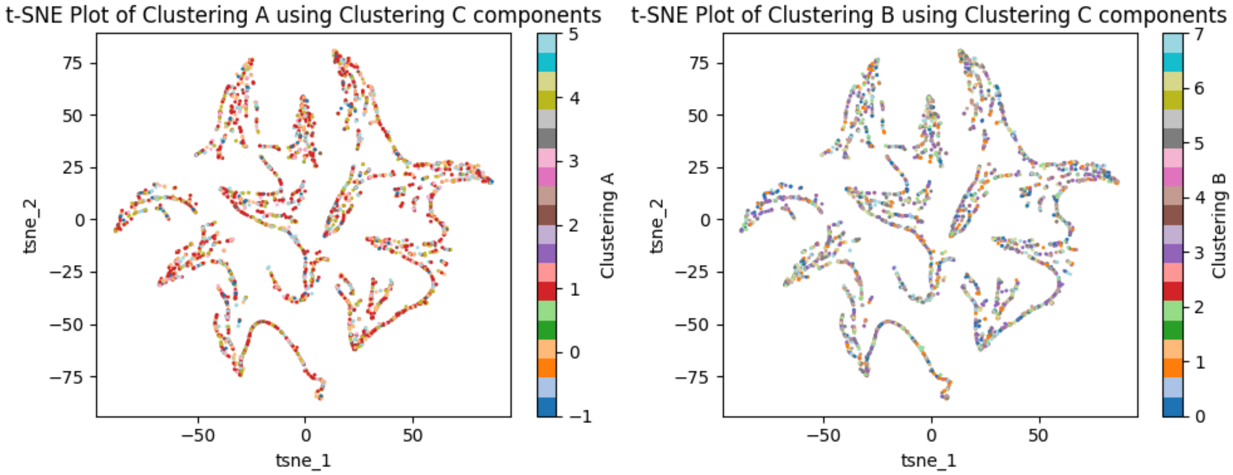

Figure D4: Clusterings A and B projected onto the tSNE components produced from the probabilistic output of Method C (described in detail in Supplemental Note C).
